# ﻿Taxonomic revision of the native *Magnolia* (Magnoliaceae) species of Brazil

**DOI:** 10.3897/phytokeys.238.113277

**Published:** 2024-02-01

**Authors:** Juliana Cruz Jardim Barbosa, Maria Beatriz Rossi Caruzo, Ana Rita G. Simões, Marie-Stéphanie Samain

**Affiliations:** 1 Instituto de Pesquisas Ambientais, Unidade Jardim Botânico, Avenida Miguel Stéfano, 3687, Água Funda, CEP 04301902, São Paulo, São Paulo, Brazil Instituto de Pesquisas Ambientais São Paulo Brazil; 2 Universidade Federal de São Paulo (UNIFESP), Instituto de Ciências Ambientais, Químicas e Farmacêuticas, Departamento de Ecologia e Biologia Evolutiva, Rua Prof. Arthur Riedel, 275 Eldorado, CEP 09972-270, Diadema, São Paulo, Brazil Universidade Federal de São Paulo (UNIFESP) São Paulo Brazil; 3 Royal Botanic Gardens, Kew, TW9 3AE, Richmond, London, UK Royal Botanic Gardens London United Kingdom; 4 Instituto de Ecología, A.C., Centro Regional del Bajío, Red de Diversidad Biológica del Occidente Mexicano, Avenida Lázaro Cárdenas 253, 61600 Pátzcuaro, Michoacán, Mexico Instituto de Ecología, A.C., Centro Regional del Bajío Pátzcuaro Mexico

**Keywords:** Brazilian Flora, conservation, distribution, Magnolioideae, Neotropics, Pinha-do-brejo, sect. *Talauma*, taxonomy

## Abstract

The genus *Magnolia* (Magnoliaceae) has a wide and disjunct geographic distribution ranging from Eastern and South Asia to Malaysia, extending across the Neartics and reaching into the Neotropics. Regarding its infrageneric classification, the genus is divided into three subgenera: *Yulania*, *Gynopodium* and *Magnolia*, the latter including the section Talauma in which the native Brazilian taxa are classified. The species of Magnoliasect.Talauma can be recognized by two parallel longitudinal scars on the petiole formed by the shedding of the stipules, in addition to a woody syncarp that breaks into irregular plates at dehiscence. Currently, in Brazil, species recognition is not clear on national platforms that are widely used by the Brazilian botanical community (e.g. Flora do Brasil), with only two native *Magnolia* species being accepted: *M.amazonica* and *M.ovata*. The lack of knowledge about the species and their respective characteristics has resulted in many identification errors in Brazilian herbaria, which contributes to the lack of knowledge about their current conservation status. We conducted a complete taxonomic revision based on extensive fieldwork, a herbarium survey, along with literature study. Based on this, we propose to recognize three previously described species, supporting the acceptance of five native *Magnolias* occurring in Brazil, namely: *M.amazonica*, *M.brasiliensis*, *M.irwiniana*, *M.ovata* and *M.sellowiana*. However, we follow the Flora do Brasil in maintaining *M.paranaensis* as a synonym of *M.ovata*. Additionally, we designate a lectotype for *M.sellowiana*. We present morphological descriptions and the geographic distribution for each species, in addition to an identification key to all of these plus the two introduced ornamental species from Asia and North America, illustrations, photographs, ecological data, updated conservation status and taxonomic notes.

## ﻿Introduction

*Magnolia* L. is a genus of approximately 370 species distributed disjunctly ranging from Eastern and South Asia to Malaysia, extending across the Neartics (Canada and USA) and reaching into the Neotropics (Stevens 2001; Aldaba Núñez et al., unpublished data). *Magnolia* species have an important ornamental value due to their colorful and showy ﬂowers (e.g., *Magnoliagrandiﬂora* L., *M.liliiﬂora* Desr., M.×soulangeana Soul.‐Bod., *M.virginiana* L., *M.ovata* (A.St.-Hil.) Spreng.), and some species have also been used in traditional medicine (e.g., *M.dealbata* Zucc., *M.liliiﬂora*, *M.mexicana* DC. and *M.officinalis* Rehder & E.H.Wilson) or as a timber species (e.g., *Magnoliadixonii* (Little) Govaerts, *M.grandiﬂora*, *M.striatifolia* Little) (Pérez-Castañeda 2015; Wang et al. 2020; Xie et al. 2022).

Despite its scientific and economic relevance, the taxonomy of *Magnolia* is incomplete, and so far, few in-depth studies have been conducted to understand all variation in its morphological characters, despite the recognition of moderate phenotypic plasticity in Magnoliaceae (Vázquez-García et al. 2014; Gutiérrez-Lozano et al. 2021). Among the studies conducted within the genus, those combining morphological and molecular data to compare individuals of widely distributed species stand out (e.g. Arteaga-Rios et al. 2020). Such studies highlight significant morphological variations and mostly conclude that different species may be confused as a single widely conceptualized species, with a large variation in occurrence and morphology (Arteaga-Rios et al. 2020). This shows the need for further research to clarify the taxonomy of this genus, especially regarding species delimitation.

**Figure 1.**
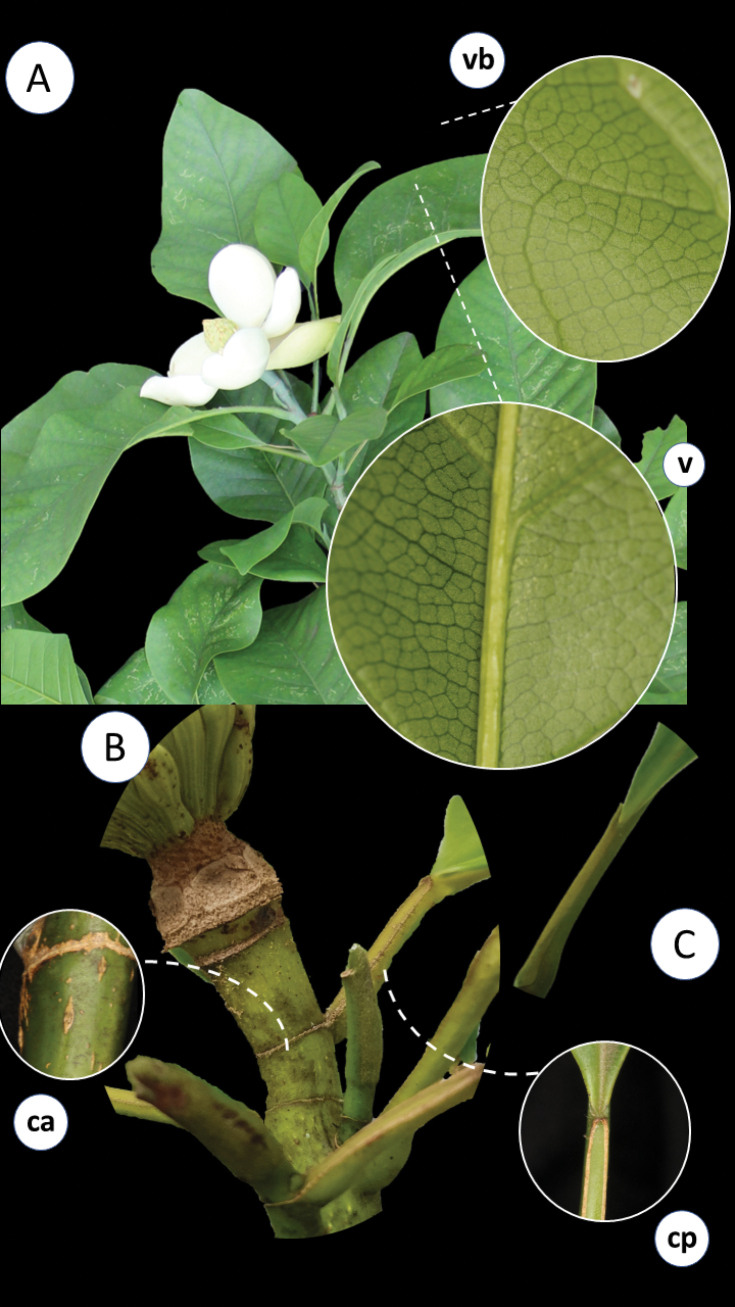
**A** branch with alternate leaves, present stipules, and terminal flower, **v.** detail of central vein, **vb.** detail of brochidodromous venation **B** detail of the branch showing scars **C** adnation of the stipule (which later falls) on the petiole, a characteristic of MagnoliasectionTalauma; **ca**. Annular scar; **cp.** Petiolar scar (resulting from stipule fall). Photos: A, v, vb, ca, cp: D.A. Zavatin; B-C: J. C. J. Barbosa.

**Figure 2.**
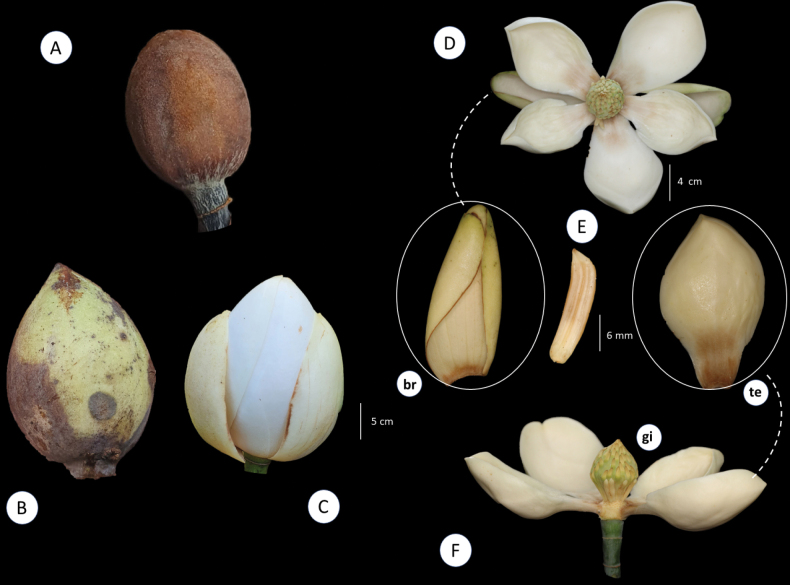
*Magnolia* flower stages **A** perule **B** immature floral bud **C** mature floral buds **D, F** flower at anthesis; **br.** Sepaloid tepal **te.** Petaloid tepal; **gi.** Flower with detail of the gynoecium **E** stamen. Photos: A: *U. Pastore & R.M. Klein 145* (MBM115080); B-C: J. C. J. Barbosa; D, bra, te: D. A. Zavatin.

**Figure 3.**
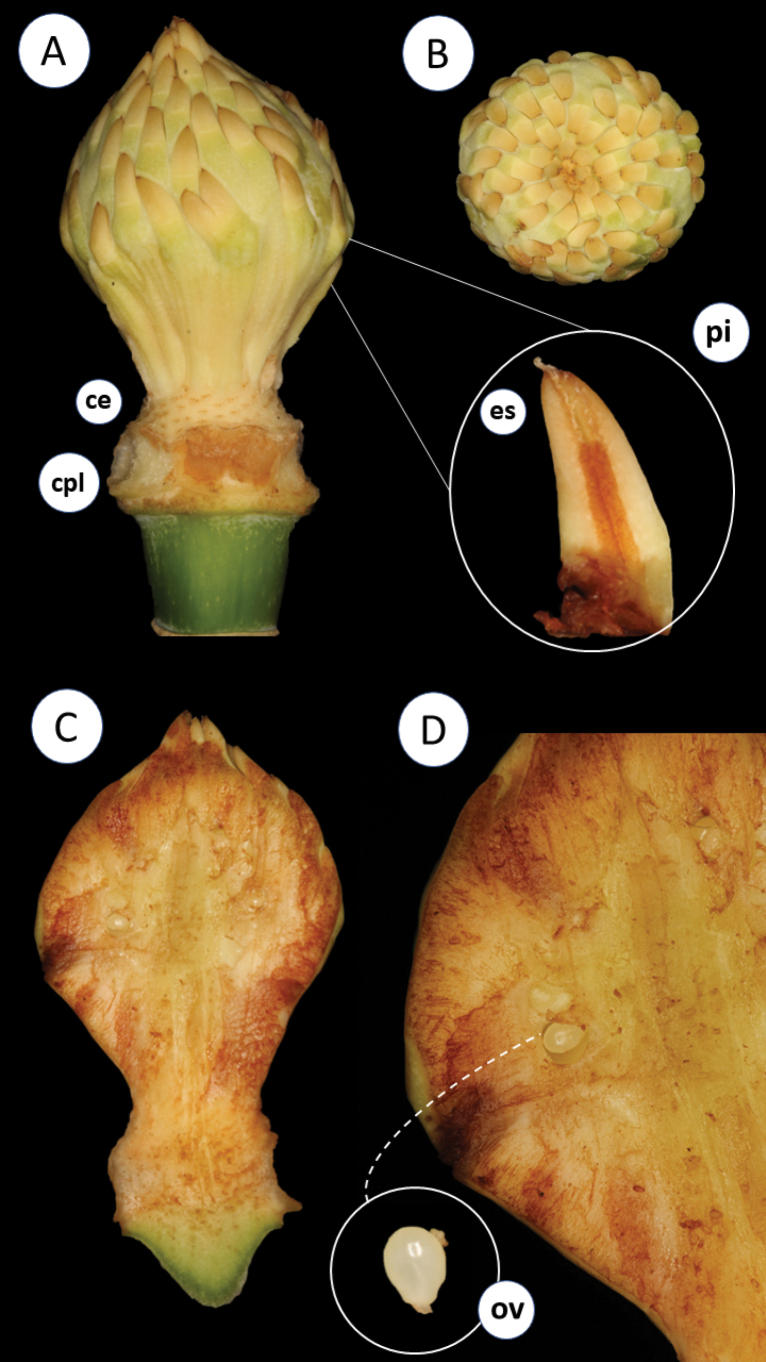
**A** gynoecium, vertical view **B** gynoecium, viewed from above **ce.** Staminal scar, **cpl.** petaloid scar, **pi.** Pistillum, **es.** Stigma **C** longitudinal section, receptacle and gynoecium **D** detail ovary, **ov.** ovule. Photos: D. A. Zavatin.

**Figure 4.**
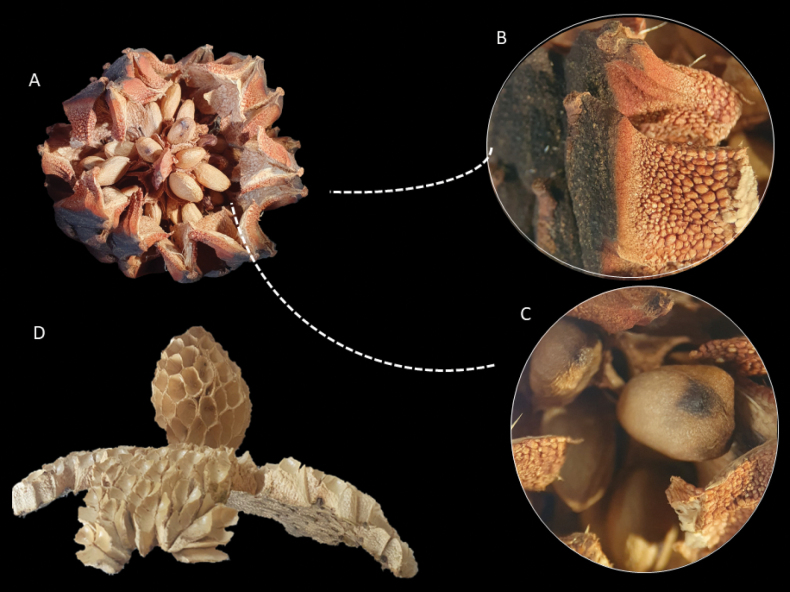
Fruit stages **A** partially open syncarpous fruit **B** detail of the inner woody part of the fruit **C** 1–2 seeds per carpel **D** mature fruit, with open woody masses (seeds already fallen from the fruit) Photos: J. C. J Barbosa.

Regarding its infrageneric classification, *Magnolia* is divided into three subgenera (Figlar and Nooteboom 2004) (subgen. Magnolia, subgen. Yulania (Spach) Rchb. and subgen. Gynopodium (Dandy) Figlar and Noot.) and 15 sections (Wang et al. 2020). Magnoliasect.Talauma belongs to the subgenus Magnolia and is the richest section in terms of species number, with nearly 130 taxa (Pérez-Castañeda 2015; Aldaba Núñez et al., unpublished data). All native Magnolia species occurring in Brazil belong to section TalaumasubsectionTalauma. Its species are characterized as perennial trees with stomata grouped in numbers of two, three, or five (Figlar and Nooteboom 2004; Wang et al. 2020). The stipules are fused to the petiole, leaving two parallel longitudinal scars after shedding (Fig. 1). The flowers are terminal, protected by one or two bracts, here referred to as ‘perula’ (Treseder 1978) (Fig. 2), with three sepals, six or seven petals, usually white or yellow, thick and fleshy. In a considerable number of species, the sepals and petals are not differentiated and are named tepals (Beentje 2010). The androecium has 20–220 stamens, and the carpels in the gynoecium can be few or numerous, free or, predominantly in South American species, fused, with each carpel having two ovules (Fig. 3). The fruit is apocarpous, multifollicular, or, in the South American species, a woody syncarp that splits into irregular plates upon dehiscence, exposing seeds with reddish or orange sarcotesta, which are individually pendulous by a funiculus (Fig. 4) (Law 1984; Lozano-Contreras 1990; Vázquez-García et al. 2016; Mello-Silva et al. 2023).

Eichler (1864) recognized two species of Magnoliaceae for Brazil: *Talaumaovata* A.St.-Hil. and *T.dubia* Eichler. Lozano-Contreras (1990), in a more comprehensive study on the family in Brazil, recognized four species for the country: *T.amazonica* Ducke, *T.irwiniana* Lozano, *T.ovata* (synonymizing *T.dubia*, due to their similarity in their leaf blade shape, number of carpels, syncarp morphology and because they do not present an indument on any of their structures), and *T.sellowiana* A.St.-Hil. Vázquez-García et. al (2013) described *Magnoliaparanaensis* as a new species for Brazil, based on new records, restricted to the Serra do Mar (Paraná). de Azevedo et al. (2018) described *M.brasiliensis* C.O. Azevedo, A.F.P. Machado & A. Vázquez, based on material collected in the states of Bahia and Minas Gerais, which were the first records of Magnoliaceae for the Northeast region of Brazil up to that point. However, considering a broader concept of species, the authors of the Flora do Brasil 2023 treatment (Mello Silva et al. 2023) only accepted four species: *M.amazonica* (Ducke) Govaerts and *M.ovata*, as native to the country, and *M.champaca* L. and *M.grandiflora* L., both introduced and cultivated. They synonymized *M.brasiliensis* with *M.amazonica*, and *M.irwiniana*, *M.paranaensis*, and *M.sellowiana* with *M.ovata*, without further explications. In the “Magnoliaceae Red List” (Rivers et al. 2016), as well as on platforms where the accepted names of *Magnolia* for Brazil are found, such as SpeciesLink (2023)SpeciesLink (2023), there are differences in species determinations, which evidences divergence in their delimitation among different authors, causing confusion in the identification of material collected throughout the country.

In 2018, Azevedo and colleagues realized that some paratypes of *M.brasiliensis*, initially identified as *M.ovata* by Pirani and De Mello-Silva (1996) in their revision of Magnoliaceae in Serra do Cipó, Minas Gerais, did not provide information on important characters that could help distinguish the species, such as the number of stamens and carpels. Although *M.ovata* is included as one of the species within the section Talauma in Wang et al. (2020), the specimen used for this phylogenetic analysis was *R. Mello-Silva et al. 50* (US), the same material used by de Azevedo et al. (2018) to describe *M.brasiliensis*. This is a good example of the widespread misidentification of distinct species as *M.ovata*.

The difficulties in delimiting the species that are addressed in this work not only affect the taxonomic scope, where identification errors are leading to wrong interpretations in broad studies, but also have consequences for the assessment of the conservation status of the taxa involved. With only two species without conservation problems, while the rest are Endangered or Data Deficient, conservation actions are urgently needed. The conservation status of the Brazilian *Magnolia* species, mentioned by Rivers et al. (2016), were assessed as follows: *M.amazonica*: Least Concern (LC); *M.irwiniana*: Endangered (EN); *M.ovata*: Least Concern (LC); *M.paranaensis*: Data Deficient (DD); and *M.sellowiana*: Data Deficient (DD). In 2021, Lamarche & Azevedo assessed *M.brasiliensis* as Endangered (EN) in the International Union for Conservation of Nature (IUCN) Red List (2022).

Taxonomic disagreements, such as the delimitation of species and the number of accepted names by different authors, have a significant impact on our understanding of the actual distribution and current conservation status of *Magnolia* species, especially regarding the supposedly widely distributed *M.ovata*. This study aims to expand the taxonomic knowledge of *Magnolia* in Brazil, focusing on the native species, and contribute to the conservation of the genus’ diversity in the Neotropical region.

## ﻿Materials and methods

To perform the analysis of botanical material, protologues and images from type collections in virtual databases such as Tropicos (2023) and JSTOR (2023) were consulted, in addition to visits to seven herbaria (HEPH, HUFSP, RB, SP, VIC, SPSF, MBM, ESA) (Thiers continuously updated). A total of approximately 100 specimens were analyzed physically, supplemented by field observations of species’ populations in their natural habitats, and approximately 180 specimens were studied in databases such as Flora e Funga do Brasil (2023) and SpeciesLink network (2023), provenant from 24 herbaria (CEN, ESA, F, FURB, G, HEPH, HJ, HUEFS, IAN, ICN, JOI, MBM, MO, NY, P, RB, S, SP, SPSF, UB, UEC, UPCB, US, VIC) (Thiers , continuously updated). All material collected during fieldwork was herborized following traditional techniques as described in Mori (1989), and samples were deposited in the HUFSP and SP herbaria. Measurements were obtained from the examined specimens, considering the smallest and largest structure analyzed, when available. In addition, tables with morphological characters (e.g., leaf shape, petiole scars, pubescence, fruits), protologue descriptions, the reference work by Lozano-Contreras (1990), and observations made, were used to complement the descriptions of each species and for further understanding the genus, such as the study of ecology and distribution. Definitions of botanical characters and terms were taken and adapted from Radford et al. (1974), Howard (1948) and Lozano-Contreras (1990).

A database of distribution records was constructed from specimens with confirmed identifications by the first author of this paper, with additional records extracted from the Flora e Funga do Brasil (2023) and SpeciesLink network (2023) databases, where the species identification was possible based on morphological and distributional information. Distribution maps were produced using QGIS software v. 3.28.3 (QGIS Development Team 2015). The Brazilian regions mentioned in the work follow those of the IBGE (2017) on the regional divisions and subdivisions of Brazil.

Geospatial analyses were conducted to determine the Area of Occupancy (AOO) and the Extent of Occurrence (EOO) using the online Geospatial Conservation Assessment Tool (GeoCAT) software (Bachman et al. 2011) (http://geocat.kew.org/editor). The IUCN categories and criteria (2022) were used to assess the preliminary conservation status for each of the species studied.

## ﻿Results

Seventeen morphological characteristics were obtained for analysis and preparing the descriptions and the identification key. Ten locations were visited in the Southeast, South and Central-West regions of Brazil.

Five species of *Magnolia* native to Brazil are here recognized: *M.amazonica*, *M.brasiliensis*, *M.irwiniana*, *M.ovata* and *M.sellowiana*, whereas *M.paranaensis* is considered a synonym of *M.ovata*. Most species occur in riparian forests and rainforest, with the exception of *M.brasiliensis*, which is found in semi-deciduous seasonal forest (Rizzini 1979).

### ﻿Taxonomic treatment

#### ﻿ Magnolia

Taxon classificationPlantaeMagnolialesMagnoliaceae

L.

AB3DD8B0-0E05-52AF-939F-C310F8D33B4F

##### Type.

*Magnoliavirginiana* L.

##### Description.

Trees or shrubs, evergreen or deciduous, branches lenticulate with internodes marked by annular scars, stipules free or, in Neotropical species (*Magnoliasect.Talauma* and *Macrophylla*), attached to the petiole, leaving 2 parallel longitudinal scars after shedding. Flowers terminal, solitary, protected by 1–2 bracts (perula); sepals 3; petals 3–12(–15), fleshy, cream-colored; stamens 20–220; carpels few to numerous, free or, predominantly in South American species, coalescent; ovules 2–(5), pollen large, > 50 μm diameter, stamens deciduous during male phase (except sect. Oyama). Fruit apocarpic, multifollicular or, predominantly in South American species, syncarpous, woody, which breaks into irregular plates at dehiscence, exposing the seeds provided with reddish or orange sarcotesta, which are individually pendulous by a funiculus (Figs 1–4) (Lozano-Contreras 1990; Figlar and Nooteboom 2004; Vázquez-García et al. 2016; Mello-Silva et al. 2023).

##### Distribution and habitat.

*Magnolias* tend to occur at higher altitudes, mostly in high and humid forests. Preference and resistance in environments with varying temperatures and precipitation depend on the species (Song et al. 2019; Aldaba Núñez et al., unpublished data). Its distribution ranges from Eastern and South Asia to Malaysia, extending across the Neartics (Canada and USA) and reaching into the Neotropics (Stevens 2001; Aldaba Núñez et al., unpublished data). In Brazil, it is found in North, Northeast, Central-West, Southeast and South regions, at elevations approximately ranging between 200 and 1400 m. It occurs in anthropized areas, riparian forest, ‘terra firme’ forest, ‘várzea’ forest, and rainforest (Rizzini 1979; Eiten 1983).

We here provide an identification key to distinguish all Brazilian *Magnolias*, including both native and cultivated species.

### ﻿Key to sections of the genus *Magnolia* in Brazil

**Table:** 

1	Flower terminal, anther dehiscence introrse	**2**
–	Flower pseudo-axillary, anther dehiscence latrorse	**sect. Michelia**
2	Stipular scar covering the entire petiole, fruit globose to ellipsoidal	**sect. Talauma**
–	Stipular scar covering a small area of the petiole, fruit ovoid	**sect. Magnolia**

### ﻿Key to species of *Magnolia* in Brazil

**Table:** 

1	Predominant branching pattern syllepsis, stamens during the male phase shedding	**2**
–	Predominant branching pattern prolepsis, stamens during the male phase persistent	***M.champaca* (introduced and cultivated)**
2	Fruit ovoid, stamens pubescent	***M.grandiflora* (introduced and cultivated)**
–	Fruit globose to ellipsoidal, stamens glabrous	**3**
3	Branches pubescent	**4**
–	Branches glabrous	**5**
4	Leaf margins entire, symmetrical	**6**
–	Leaf margins sinuate, asymmetrical	***M.irwiniana***
5	Fruits strigose, leaves coriaceous	***M.brasiliensis***
–	Fruits glabrous, leaves papyraceous	***M.ovata***
6	Leaves broadly elliptic, secondary veins 5–13 pairs, carpels ca. 100	***M.sellowiana***
–	Leaves elliptic, secondary veins 8–19 pairs, carpels ca. 46	***M.amazonica***

#### ﻿ Magnolia amazonica

Taxon classificationPlantaeMagnolialesMagnoliaceae

(Ducke) Govaerts, World Checkl. Bibliogr. Magnoliaceae, 70. 1996.

E55CC925-B057-50BD-ACF0-A6B84DC878EA

Figs 5 , 6 , 11

≡ Talaumaamazonica Ducke, Arch. Jard. Bot. Rio de Janeiro 4: 11. 1925.

##### Type.

Brasil. “Prope medium flumen Tapajoz civitatis Pará loco Francez”, fl, 10 January 1922, *A. Ducke 12487* (holotype: RB! [RB00540679]; isotypes: B! [B10 0248229],BM! [BM000551380, BM000551379], G! [G00352605], K! [K000470024, K000470025], P! [P00734783], R! [R000024142], RB! [RB00556527, RB00556528], S! [SR6051]).

**Figure 5.**
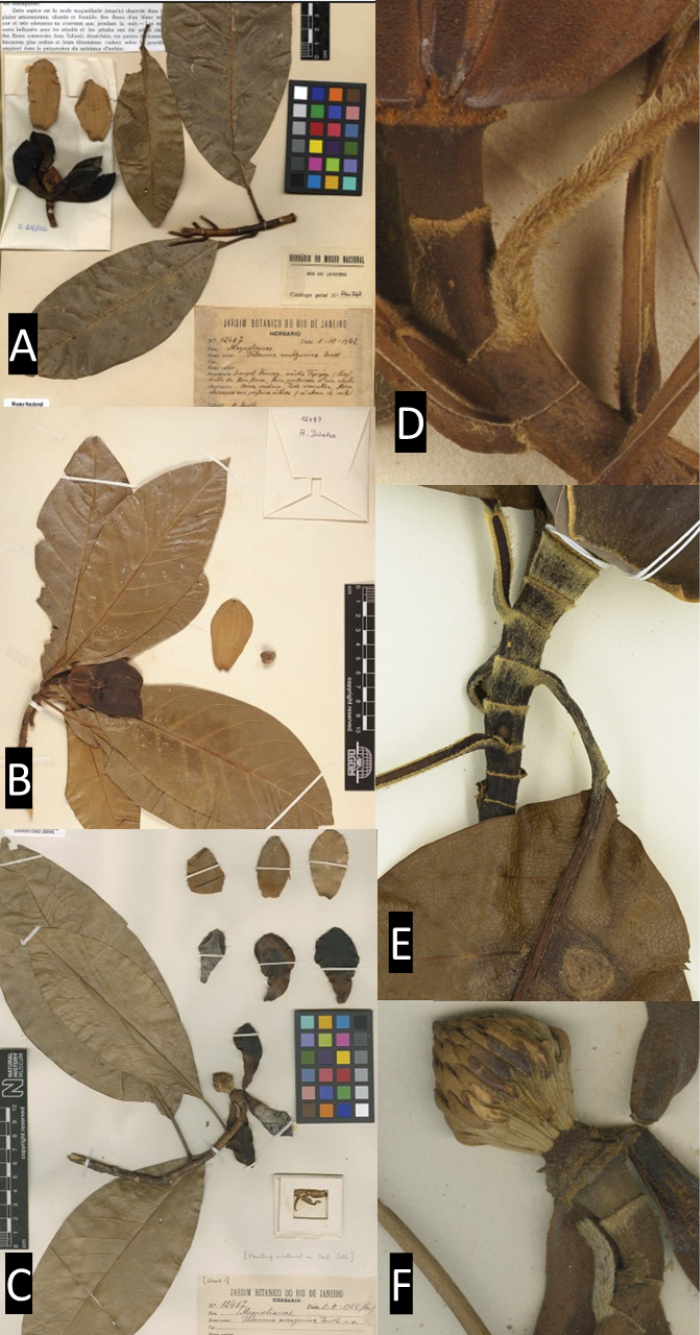
*Magnoliaamazonica***A–C** specimen deposited in herbarium **D–E** detail petiole and peduncle (in the region of the annular scars) showing trichomes in the youngest structures **F** gynoecium. Photos: **A–C***W.A. Ducke 12487* (R000024142; BM000551380); **B–D**: (B100248229); **C–F**: BM551380; **F**: *I. M. Silva 471* NY 03097880.

**Figure 6.**
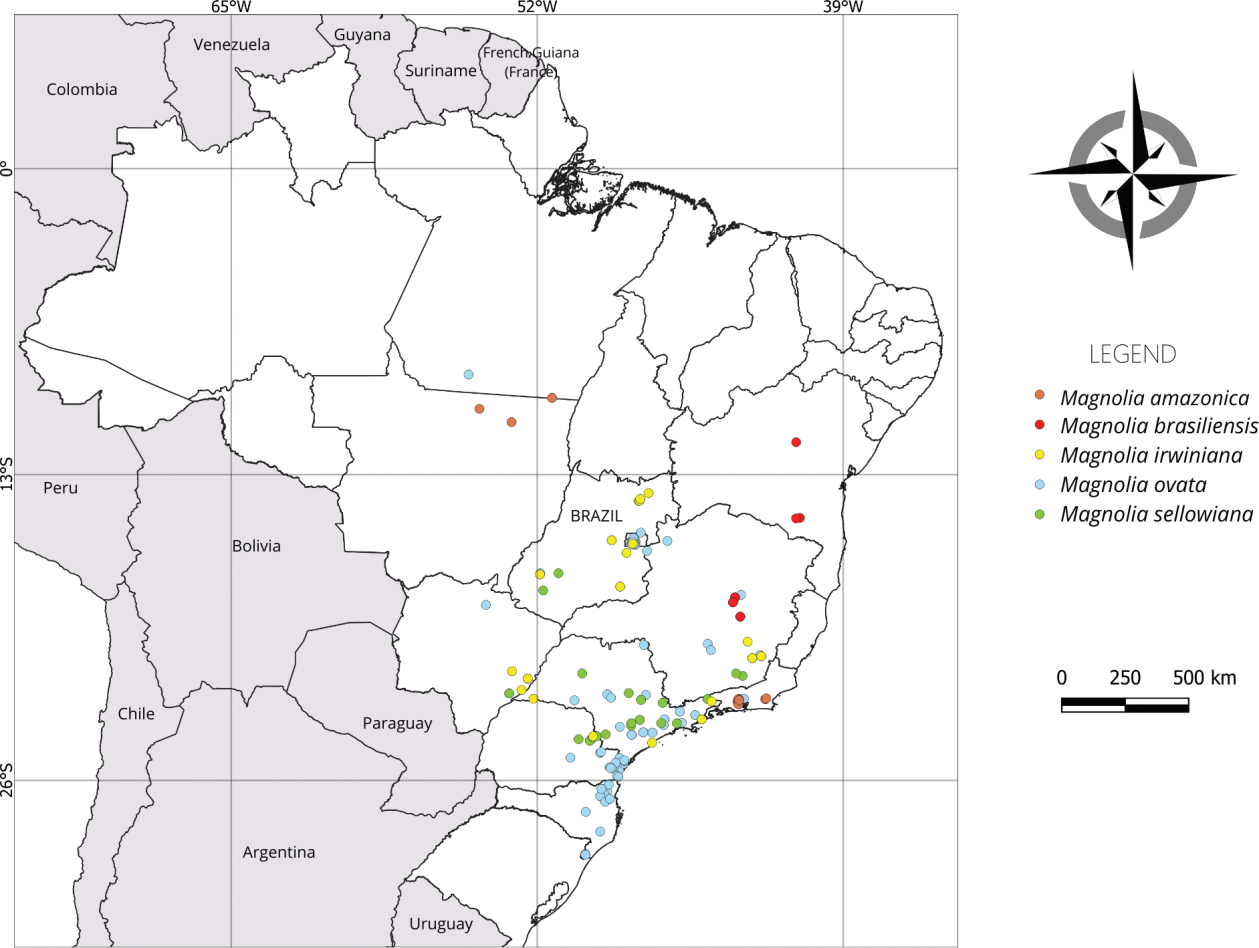
Geographical distribution of *Magnolia* species in Brazil.

##### Description.

***Trees*** 15–20 m tall; ***branches*** cylindrical, yellowish-brown, lenticulate, tomentose at annular scars closest to the flower bud, trichomes yellowish. ***Stipules*** adnate to petiole, green, oblong to conical, apex obtuse, base truncate, deciduous, tomentose. ***Petioles*** 1.8–5 cm long, stipular scar ranging from 90% to 100% of its length, tomentose when young and short, trichomes glabrescent. ***Leaf blades*** 11–28 cm × 4–12.4 cm, elliptic, base cuneate to acute, apex acute, margin entire; slightly coriaceous; venation pinnate, brochidodromous, 8–19 pairs of secondary veins irregularly spaced apart; when young tomentose abaxially, adaxially glabrous, yellowish-green. ***Peduncle*** cylindrical, tomentose at the annular scars, yellow trichomes, annular scars present. ***Flowers*** terminal, solitary; ***flower bud*** 3.95–6.34 cm × 3.25–4.70 cm, ovoid, yellowish-white, glabrous, protected by perula enveloping and protecting the flower bud, perula concave, brownish when dried; ***outer sepalloid tepals*** 3, 5–7 × 3–4 cm, asymmetrical, base cuneate, apex rounded, yellowish when dry; ***inner petaloid tepals*** 6, 6–7 cm × 3–5 cm, oblong, base attenuate, apex rounded, brown when dry; ***stamens*** ca. 100, laminar, slightly falcate, spirally arranged in 4–5 series, apex obtuse, whitish to yellowish, thecae 2, anthers introrse, dehiscence longitudinal; **gynoecium** 1.97 cm × 1.78 cm, conical, yellowish, carpels ca. 46. ***Immature* fruits** 4.4–5.5 cm long, 5 cm in diameter, globose, with puberulent pubescence, dehiscence circumscissile, in irregular syncarpous masses; ***seeds*** 1–2 per carpel, sarcotesta red.

##### Distribution and habitat.

*Magnoliaamazonica* is the only Brazilian *Magnolia* known from the Amazon region. In Brazil, it is found in the North (Amazonas and Pará) and Southeast (Rio de Janeiro) regions, and it is also known from the tropical forests of Peru and Bolivia (Lozano-Contreras 1990), although other species have been recently described there, being segregated from this species, e.g. *M.peruviana* A. Vázquez. As a consequence, the presence and distribution of *M.amazonica* in that country needs further investigation. *Magnoliaamazonica* is a perennial tree that grows up to 20 m tall in Amazon rainforest.

##### Phenology.

Its creamy-white flowers open at night and were collected in mid-January. Its fruits were observed in mid-July (Ducke 1925).

##### Preliminary conservation status.

This species has previously been assessed as Least Concern (LC) (Khela 2014). However, in this analysis (Brazilian specimens) its area of occupancy (AOO) is about 44.000 km^2^ and it is considered to be Endangered (EN) B2b (i,ii) (IUCN 2022). It is likely that this species is declining due to deforestation and land use changes, especially in the northern region of the country, where unfortunately there are flawed laws regarding preservation (Gonçalves et al. 2010). In addition, with the recent description of a *Magnolia* species in its distribution area in Peru (*M.peruviana*), the delimitation of *M.amazonica* may be narrowed in the future, with further studies. Therefore, the conservation status will also likely need to be updated.

##### Specimens examined.

**Brasil. Pará**: Novo Progresso, Serra do Cachimbo, Área da Aeronáutica torre 2 do Stand de tiro, mata de transição com campinarana, solo areno-argiloso, 9°19'16"S a 9°16'196"S, 54°59'42"W a 54°56'222"W, 20 Aug 2003, *A.S.L. Silva 3967* (RB787799); **Rio de Janeiro**: Município Silva Jardim, Reserva Biológica de Poço das Antas, Trilha do Morro do Calcário, 22°30’/22°33'S, 42°15’/42°19'W, 5 Mar 1993, *S.M. Barreto 30* (RB300133); Nova Iguaçu, Margem do Brejo do Macuco, 12 Dec 2001, *S.J. Silva Neto & M.V. Pereira-Moura 1573* (RB364320); Nova Iguaçu, Região SE, Rebio, Tinguá, Estrada do Ouro, Ponto 154, Planalto próximo a entrada para Igrejinha de Santana, 600 msm, 22°33'56.9"S, 43°28'11.2"W), 25 Jan 2006, *R.D. Ribeiro 569* (RB419738).

##### Notes.

*Magnoliaamazonica* is recognized by its puberulent-tomentose pubescence (on several of its structures, e.g., branches, stipules, petioles (Table 1), and can be found in the Amazon region of the country.

**Table 1.** Morphological, geographic, vegetation and phenology comparison table between native *Magnolia* species occurring in Brazil. (*from Lozano (1990); **from Azevedo et al. (2018).

	*M.amazonica*	*M.brasiliensis*	*M.irwiniana*	*M.ovata*	*M.sellowiana*
Pubescence of the peduncle	Tomentose	Glabrous	Glabrescent	Glabrous	Glabrescent
Presence of lenticels	Lenticulate	Densely lenticulate	Sparse lenticels	Lenticulate	Sparse lenticels
Petiole size (cm)	1,8–5	1,8–3,8	2,3–6	2,5–5 cm	2–5,6
Pubescence of the petiole	Tomentose	Glabrous	Glabrescent	Glabrous	Glabrescent
Leaf size (cm)	11 – 28 × 4 – 12	7,5–15,2 × 3,5–7,1	9-19 × 5-9,2	12,7–29,07 × 7,8 – 16,5	10-15,1 × 4,7-10
Leaf shape	Elliptic	Elliptic to oval	Oval-elliptic	Ovate-elliptic	Broadly elliptic
Leaf margin	Entire	Entire	Undulate	Entire	entire-irregular
Pairs of secondary veins	8–19	8–12	6–11	8–13	5–13
Leaf texture	Slightly coriaceous	Strongly coriaceous	Slightly coriaceous	Papyraceous	Papyraceous-membranous
Pubescence of the leaf	Sericeous- tomentose	Glabrous	Glabrescent	Glabrous	Glabrescent
Petaloid tepal size	Petaloid 6, 6–7 cm × 3–5 cm	Petaloid 6(7), 3–3,5 cm × 1,3–1,7 cm	Petaloid 6, 3.0–3.8 cm × 2.4–3.2 cm	Petaloid 6, 3,0–3,8 cm × 2,4–3,2 cm	Petaloid 6, 2,7–3,1 cm × 1,5–2,9 cm
Sepaloid tepals size	Sepaloid 3, 5–7 cm × 3–4 cm	Sepaloid 3, 3–3,2 × 2,4–3,2 cm **	Sepaloid 3, 4.5–4.8 cm × 3.5–3.8 cm	Sepaloid 3, 4,5–4,8 cm × 3,5 – 3,8 cm	Sepaloid 3, 3,4–4,0 cm × 2,7–3,2 cm
Number of stamens	98–102**	75–93**	ca. 114*	144–150*	ca. 180*
Pubescence of the fruit	Puberulent	Strigose short	Puberulent	Glabrous	Glabrous
Number of carpels	44–48	40–57**	111*	68–71*	102*
Distribution	Amazon region (Brazil, Bolivia and Peru), Southeast Region (Rio de Janeiro)	Endemic Northeast Region (Bahia) Southeast Region (Minas Gerais)	Endemic Southeast (São Paulo, Minas Gerais), and Central-West (Distrito Federal,Goiás, Mato Grosso do Sul) regions	Endemic North, South, Southeast, Midwest	Endemic Southeast (São Paulo, Minas Gerais), and Center-West (Goiás, Mato Grosso do Sul)
Vegetation	Tropical forest	Bahia: Semi-deciduous seasonal forest; Minas Gerais: Associated with watercourses and riparian forests	Tropical deciduous and riparian forests (next to watercourses)	Riparian forest and montane rain forest	Riparian forest
Phenology	Flowers: Mid-January	Flowers: October and December	Flowers: October to January	Flowers: September to December	Flowers: March to December
	Fruits: Mid-July	Fruits: January to March	Immature fruits: mid-October to March	Immature fruits: March to October	Immature fruits: January to July
				Mature fruits: June to September	

The specimen *A.S.L. Silva 3967* in the herbarium of the Botanical Garden of Rio de Janeiro (RB787799) had been erroneously identified as *M.ovata*, likely because of the similarity in the leaf shape between both species. However, they can be differentiated by the absence of trichomes in *M.ovata* (vs. trichomes present on petiole and branches in *M.amazonica*) and the number of carpels: 144–150 in *M.ovata* vs. 98–102 in *M.amazonica*.

#### ﻿ Magnolia brasiliensis

Taxon classificationPlantaeMagnolialesMagnoliaceae

C. O. Azevedo, A. F. P. Machado & A. Vázquez, Brittonia 70(3): 307. 2018.

DFE8D1A4-4A52-5EB1-9008-DAAB0C929320

Figs 6 , 7

≡ Talaumabrasiliensis (C.O.Azevedo, A.F.P.Machado & A.Vázquez) Sima & Hong Yu, J. W. China Forest. Sci 49(4): 34 2020.

##### Type.

Brasil. Bahia: Vitória da Conquista, Poço Escuro, 14°52'S, 41°0'W, 900–1300 m, fl., 10 November 2008, *C. O. Azevedo et al. 354* (holotype: HUEFS! [HUEFS000037437]).

**Figure 7.**
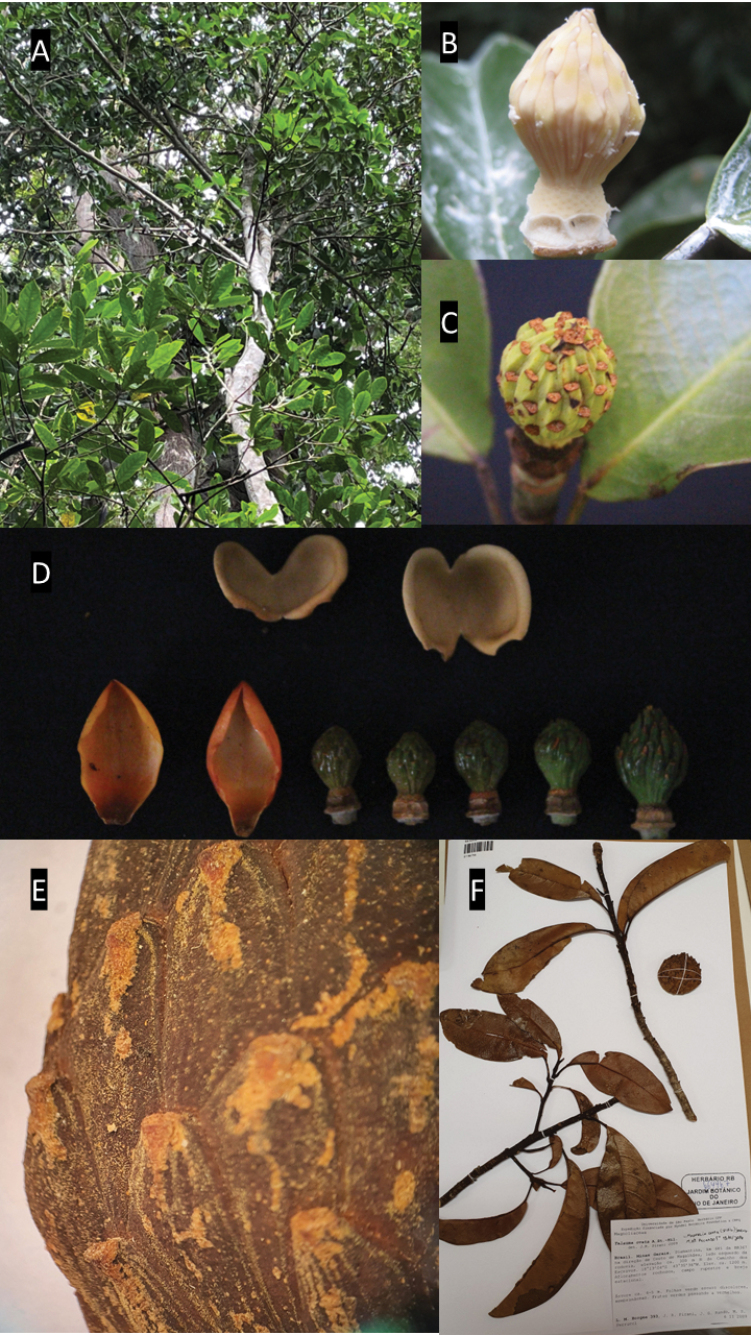
*Magnoliabrasiliensis***A** habit **B** immature gynoecium **C** immature fruit **D** bracts (perule) and gynoecium **E** details of trichomes on the fruit **F** specimen deposited in the RB herbarium showing coriaceous leaves. Photos: **A–D**: C. O. Azevedo; **E**: *R. Mello-Silva 50* (RB409806); **F**: *L.M. Borges 393* (RB664467).

##### Description.

***Trees*** 10–20 m tall; ***branches*** cylindrical, blackish when dried, with sparse lenticels, glabrous. ***Stipules*** adnate to petiole, 4–5 mm long, green, oblong to conical, apex obtuse, base truncate, deciduous, glabrous. ***Petioles*** 1.8–3.8 cm long, stipular scar along their entire length (100%), glabrous. ***Leaf blades*** 7.5–15.2 cm × 3.5–7.1 cm, elliptic to oval, base acute, apex acute to obtuse, margin entire, strongly coriaceous when dried, venation pinnate, brochidodromous, 8–12 pairs of secondary veins, glabrous, prominent on both faces. ***Peduncle*** cylindrical, glabrous, annular scars present. ***Flowers*** terminal, solitary, ***flower bud*** ellipsoid, 3–4 × 2–2.5 cm; protected by perula enveloping and protecting the flower bud, perula concave, green to yellowish when mature, brownish when dried; ***outer sepaloid tepals*** 3, 3–3.2 cm × 2.4–3.2 cm, navicular, obovate, base truncate, apex rounded, greenish; ***inner petaloid tepals*** 6 (7), 3–3.5 cm × 1.3–1.7 cm, navicular, spathulate, apex obtuse, base attenuate to truncate, cream-colored; ***stamens*** 75–93, 8–9 mm, laminar, slightly falcate, arranged spirally in 4–5 series, apex obtuse, whitish to yellowish, thecae 2, anthers introrse, dehiscence longitudinal; ***gynoecium*** 1.8–2 cm × 1–1.3 cm, conical to ellipsoid, cream-colored, slightly suberous, carpels 40–57. ***Immature fruits*** 4.4–6.7 cm long, 5 cm in diameter, obovoid to broadly ovoid, occasionally subspherical, cream-green basally, dark green distally, lenticellate, with short yellowish strigose trichomes; ***mature fruits*** 7–8 cm × 6–7 cm subspherical, dehiscence circumscissile, in irregular, blackish syncarpous masses; ***carpels*** slightly prominent, blackish on dorsal wall; ***seeds*** 1–2 per carpel, angular, obovoid, 8–12 mm long, 5 mm thick (broadest side), sarcotesta dark red, scented.

**Figure 8.**
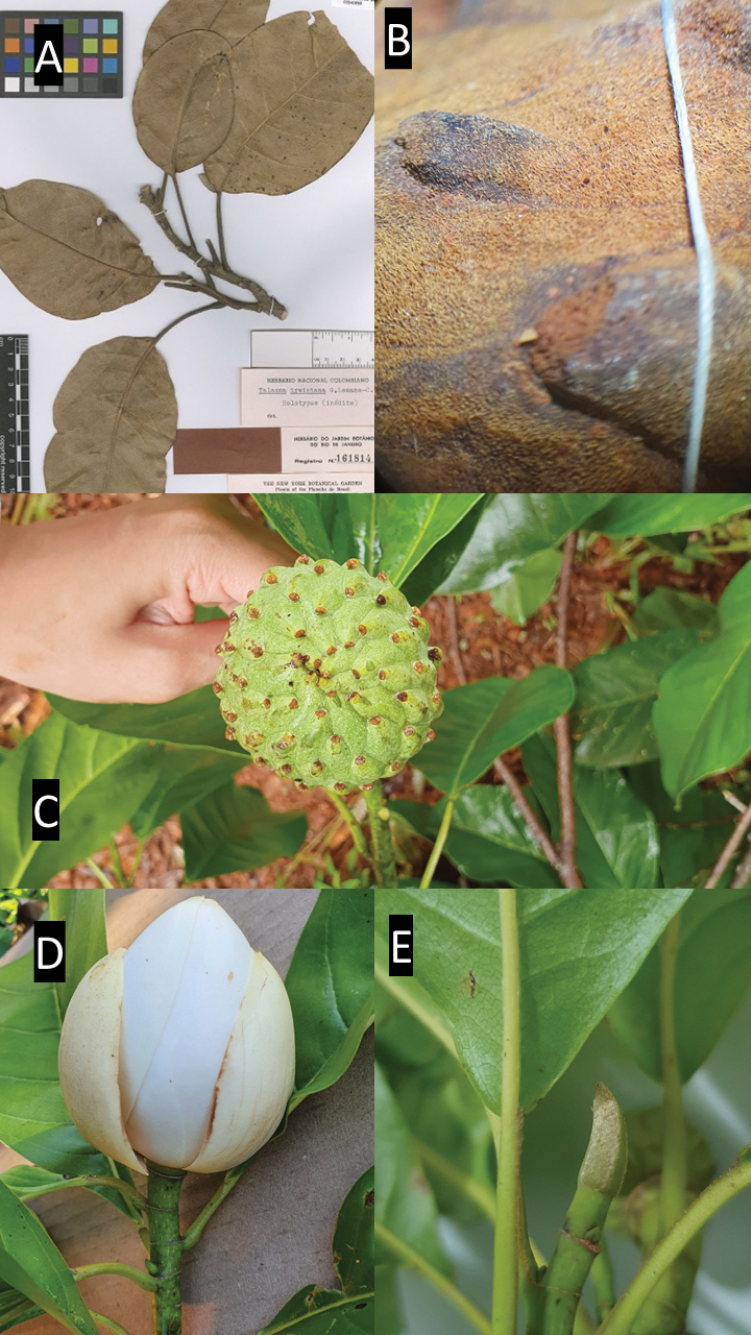
*Magnoliairwiniana***A** herbarium specimen, asymmetrical leaves **B** detail of trichomes on the carpels **C** immature fruit **D** floral bud **E** detail of stipule and petiole scar. Photos: **A**: *H.S Irwin 12681* (RB 540686); **B**: *H.S Irwin 12681* (MO 216832) **C–E**: *J. C. J. Barbosa*.

##### Distribution and habitat.

*Magnoliabrasiliensis* is an endemic species that has been found in the states of Bahia and Minas Gerais, typically at 900–1300 m elevation (de Azevedo et al. 2018). In Bahia, it occurs in Mata de Cipó, in semi-deciduous seasonal forest, in the transition between Caatinga and Atlantic Forest. In Serra do Espinhaço, in Minas Gerais, *M.brasiliensis* is always associated with watercourses and riparian forests (de Azevedo et al. 2018).

##### Phenology.

The species was observed flowering between October and December and fruiting between January and March.

##### Preliminary conservation status.

This species has been assessed as Endangered (EN) (Lamarche and de Azevedo, 2021), which is confirmed in this analysis, despite a few additional records. The area of occupancy (AOO) is about 24.000 km^2^ and it is thus considered to be Endangered (EN) B2b (i,ii) (IUCN 2022), mainly taking into account its low occurrence number in current localities, and the possible risk of degradation of its natural habitat in the state of Bahia.

##### Specimens examined.

**Brasil. Bahia**: Morro do Chapéu, Rio Ferro Doido, 22 km L de Morro do Chapéu, 01 May 1999, *F. França 2780* (HUEFS37437); Vitória da Conquista, Chapada dos Cactos, Poço Escuro, 10 Nov 2008, *C. Acevedo 354* (HUEFS145909); **Minas Gerais**: Conceição do Mato Dentro, Serra do Cipó, 13 Nov 2004, *A.E.H. Salles 3322* (HEPH12162); Ca. 7 km N.E of Diamantina, road to Mendanha, 29 Jan 1969, *H.S. Irwin 22808* (V0218886F); Morro do Coco, próximo ao trevo para Diamantina, ca. 1300 m, 18°26'S, 43°41'W, 21 Mar 1989, *R. Mello Silva 49* (MBM138963, V0218885F); Diamantina, km 685 da BR 367 na direção de Couto de Magalhães, lado esquerdo da rodovia, 18°13'04"S, 43°35'36"W, afloramentos rochosos, campo rupestre e brejo estaciona, 6 Jan 2009, *L.M. Borges 393* (CEN92706, HUEFS224097, RB664467); Mun. de Jaboticatubas, km 140 ao longo da rodovia Lagoa Santa-Conceição do Mato Dentro, 29 Feb 1980, *J.R. Pirani 5949* (SP168043); Santana do Riacho, Serra do Cipó, córrego 2 pontinhas, 24 March 1989, *R. Mello Silva 15953* (US 1483304); Serra do Cipó, córrego 2 pontinhas, ca. 1220 m, 19°85'S, 43°34'W, 24 Mar 1989, *R. Mello Silva 50* (MBM138964, RB409806, V0218888F); Serra do Espinhaço. Serra do Cipó, 18 Feb 1972 *W.R. Anderson 8935* (US1996644); Serra do Cipó, Mun. Santana do Riacho, rodovia Belo Horizonte, Conceição do Mato Dentro km 112, córrego 2 pontinhas, 1250 m, *A.A. Grillo & M. Sztutman >s.n.* (SP13861).

##### Notes.

*Magnoliabrasiliensis* is easily distinguished from other species of the genus occurring in Brazil due to its vegetative characteristics (Table 1). The species has elliptic leaves with entire margins, glabrous, coriaceous and smaller (7.5–15.2 cm × 3.5–7.1 cm) (vs. differently shaped, undented, membranous and larger leaves) when compared to other *Magnolia* species from Brazil. Another interesting character is the short strigose pubescence on its fruit, with linear distribution along its furrows, different from other species where the pubescence is broader and denser (e.g. in *M.amazonica*) (Figs 7, 11). Moreover, *M.brasiliensis* is the only representative of the genus known from Bahia.

The region where *M.brasiliensis* occurs is drier than that from the other species, in a transition area between Caatinga and Atlantic Forest of Brazil, a region that despite being humid, has a lower intensity of rainfall than other areas of the same domain, which may be a determining factor for the size and texture of the leaves and also for petiole size (Gutiérrez-Lozano et al. 2021).

#### ﻿ Magnolia irwiniana

Taxon classificationPlantaeMagnolialesMagnoliaceae

(Lozano) Govaerts, World Checkl. and Bibliogr. Magnoliaceae: 71. 1996.

9A8983B9-DDFC-5FF7-A24F-FE8E5EF0ABAF

Figs 6 , 8

≡ Talaumairwiniana Lozano, Rev. Acad. Colomb. Ci. Exact. 66: 580. 1990.

##### Type.

Brasil. Goiás: Chapada dos Veadeiros, “ca. 15 km W of Veadeiros”, 1000 m, 12 February 1966, fr., *H.S. Irwin et al. 12681* (holotype: RB! [RB00540686]; isotypes: COL!, MO! [MO216832], NY! [NY00320735, NY00320738], US! [US00433287, US00433288]).

##### Description.

***Trees*** ca. 15 m tall; ***branches*** cylindrical, with sparse lenticels, with cream-colored, tomentose and glabrescent trichomes, ***Stipules*** adnate to petiole, 0.5–1 cm long, green, oblong to conical, obtuse apex, truncate base, deciduous, tomentose when young. ***Petioles*** 2.3–6 cm long, stipule scar over its entire length (100%), yellowish-villous-tomentose trichomes when young. ***Leaf blades*** 9–19 cm × 5–9.2 cm, oval-elliptic, asymmetrical, apex obtuse-rounded, base cuneate-cordate, margin undulate, when young tomentose on abaxial side, glabrescent or trichomes persistent in herbarium material; venation pinnate, brochidodromous, abaxially slightly tomentose when young, adaxially glabrous, brown or yellowish; 6–11 pairs of secondary veins, glabrous, brown or yellowish. ***Peduncle*** cylindrical, tomentose at the annular scars, yellow trichomes or glabrescent, annular scars present. ***Flowers*** terminal, solitary, ***flower bud*** ovoid, 4.1 × 3.5 cm, white, glabrous, protected by the perula which is enclosing and protecting the flower bud, perula concave, brownish when dried; ***outer sepaloid tepals*** 3, 4,8–5 cm × 2,1–4 cm, cream-colored, navicular, spathulate, apex obtuse, base attenuate to truncate, glabrescent; ***inner petaloid tepals*** 6, 4,5–4,6 cm ×1,8–2,3 cm, navicular, spathulate, apex obtuse, base attenuate to truncate; ***stamens*** ca. 114, 1.2–1.4 cm × 0.1–0.2 mm, laminar, slightly falcate, arranged spirally in 4 series, apex obtuse, whitish to yellowish, thecae 2, introrse, dehiscence longitudinal; ***gynoecium*** conical to ellipsoid, slightly suberous, cream-colored, carpels ca. 111. ***Immature fruits*** 3–4 cm × 4.2–4.5 cm, obovoid to irregular shape, dehiscence circumscissile, in irregular syncarpous masses, yellow puberulent trichomes, ***seeds*** 1–2 per carpel.

##### Distribution and habitat.

*Magnoliairwiniana* occurs in tropical deciduous and riparian forests (next to watercourses). During collecting expeditions, it was found exactly in a saturation area, on waterlogged soil. It occurs in the Southeast (São Paulo, Minas Gerais) and Central-West (Goiás) regions.

##### Phenology.

The species was found with flowers between October and January and immature fruit was observed in mid-October and March.

##### Preliminary conservation status.

This species has been assessed as Endangered (EN) (Global Tree Specialist Group, 2014), which is here confirmed. The area of occupancy (AOO) is about 96.000 km^2^ and it is thus considered to be Endangered (EN) B2b (i,ii) (IUCN 2022). Despite having a reasonable number of locations, it was observed during expeditions that the sites where the species was found were degraded or extremely fragmented (in one case having only one adult individual in an area), which exemplifies the serious decline in habitat.

##### Specimens examined.

**Brasil. Distrito Federal**: Brasília, Reserva ecológica do IBGE, proximidade do córrego Taquara, na divisa com Jardim Botânico de Brasília (Cristo) e Fazenda Água limpa-FAL-Unb, 13 Feb 2014, *M. Aparecida da Silva 8015* (RB1140562); **Goiás**: Alto Paraíso de Goiás, Camping Portal da Chapada, Centro Oeste, Mata de galeria, 1164 m, 11 Jan 2002 *L.H. Soares 1208* (RB534341); Chapada dos Veadeiros, gallery forest and adjacent campo. ca. 15 km. W. of Veadeiros, Goiás, 12 Fev 1966, *H.C. Irwin 12681* (MO216832, NY320735, IAN137999); Margem esquerda do lago, cerca de 1,5 km após a Barragem (montante), 30 Marc 2005, *A.A Santos 2576* (CEN66134); Near Pico dos Pirineus, 26 Jan 1968, *H. S. Irwin et al. 3734* (US2221273); Teresina de Goiás, Estrada Alto Paraíso Teresina, 10 Out 1979, *E. P. Heringer et al. 1658* (US3319311); **Mato Grosso do Sul**: Bataguassu, estrada para Anaurilândia, 19 Nov 1992, *I. Cordeiro et al. 922* (SP268180); Estrada Bataguassu-Brasilândia, próximo a Bataguassu, 22 Nov 1991, *I. Cordeiro 1030* (SP268194); **Minas Gerais**: Fazenda do Toninho, Alvinopolis, 15 Jun 1997, *C.C Paula 1393* (VIC17332); Araponga, Pq Estadual, perto de um centro de pesquisa, 05 Jan 2008, *B.S. Leoni 7072* (RB739528); Santos Drummont, Posses, Sítio Aracá, nascentes do córrego Araçá, 1000 m, 21°28'03"S, 43°39'26"W, 15 Oct 2003, *R. Mello Silva 2168* (RB 394934); Córrego Do Bárbaro, Parque Nacional da Serra da Canastra, São Roque de Minas, 19 Oct 1997, *J.N. Nakajima 2990* (ESA102608); Conceição do Mato Dentro, 10 Jan 2022, *J.C.J. Barbosa et al. 14* (SP540865); Viçosa, 2 Nov 1935, *C. Baez 1662* (RB210355); Viçosa, Estação de Pesquisa, Treinamento e Educação Ambiental, Mata do Paraíso, 13 Jun 2013, *M.V.R.C Simão 326* (VIC40472); Sítio Bom Sucesso, fragmento de mata próximo de nascente de rio,24 Nov 2021, *J.C.J Barbosa & J.D.B. Miranda 11* (SP540864); **São Paulo**: Estação Ecológica Juréia-Itatins, Margens do Rio Verde, proximidades do Pocinho, 12 Marc 1992 *S. Aragaki 13* (SP253046); São José do Barreiro, Fazendo Atibaia, Acesso pelo km 258 da Rodovia dos Tropeiro, Interior da Mata do Mascote, 4 Jul 2007, *H. Serafim 276* (RB719859); Reserva Estadual do Morro do Diabo, Mun. Teodoro Sampaio (à direita do Angelim), 28 Nov 1985, *O.T Aguiar 152* (SPSF9544); Ubatuba, Praia de Itamambuca, 05 Feb 1996 *H.F Leitão Filho et al. 34821* (SP295573).

##### Notes.

*Magnoliairwiniana* has been extensively confused with *Magnoliaovata*, but it can be easily distinguished by the asymmetrical leaf with undulate margin, and the presence of trichomes on its structures (vs. symmetrical leaf with entire margin and glabrous structures) and the high number of carpels, ca. 111 (vs. 68–71) (Figs 11, 12).

#### ﻿ Magnolia ovata

Taxon classificationPlantaeMagnolialesMagnoliaceae

(A.St.-Hil.) Spreng, Syst. Veg. 4(2): 217. 1827.

58A6D4F0-BA9E-5099-8ECF-BD46EFE6F067

Figs 6 , 9

≡ TalaumaovataA.St.-Hil., Fl. Bras. Merid. 1: 26, t. 4, f. A. 1824.
= Talaumadubia Eichler, Fl. Bras. 13 (1): 126, 1864. Type. BRAZIL (W). S.l., s.d., *Pohl >s.n.* (lectotype designated here: BR! [BR5429745], isolectotype; BR5430390]).
≡ Talaumaparanaensis (A.Vázquez) Sima & Hong Yu, J. W. China Forest. Sci 49(4): 36 2020.
= MagnoliaparanaensisA.Vázquez, Recursos Forest. Occid. México 4(2): 473 2013. Type. BRASIL. Paraná: Município de Cerro Azul, estrada antiga, Cerro Azul-Jaguariavia, 12 km depois da ponte sobre o Río Ribeira, 24°45'S, 48°45'W, 7 December 1983, *R. Callejas et al. 1871* (holotype: MO! [MO1942518]; isotypes: COL, MB, NY! [NY 413243]).
non Magnoliaovata P.Parm., Bull. Sci. France Belgique 27: 193, 250 1896 ≡ Magnoliadodecapetala (Lam.) Govaerts, World Checkl. & Bibliogr. Magnoliaceae [D.G. Frodin & R. Govaerts] 70 1996.

##### Type.

Brasil. Minas Gerais: “In paludosis prope Olho d’Água, parte occidentali provinciae Minas Gerais quam vocant Certão”, fl., *St. Hilaire >s.n.* (holotype: P! [P00734790], isotypes: MPU! [MPU027385], P! [P00734791, P00734792]).

**Figure 9.**
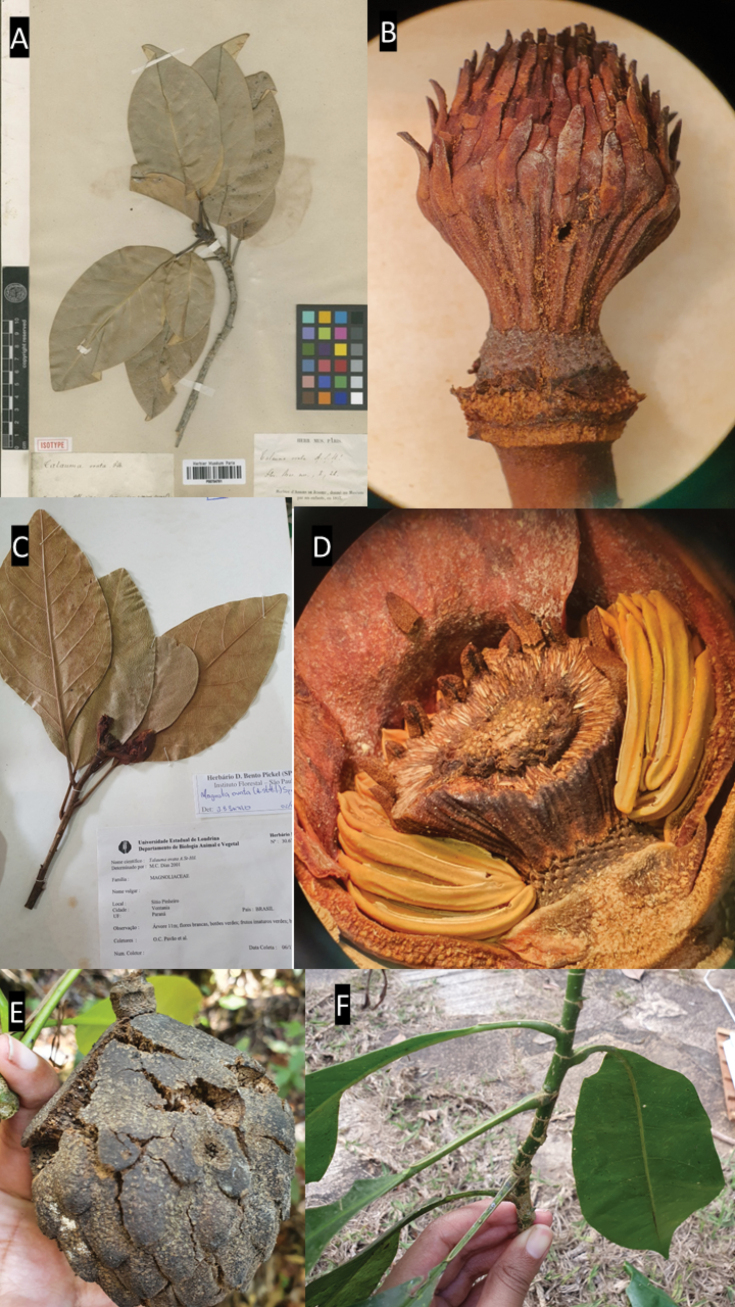
*Magnoliaovata***A** specimen deposited in herbarium P **B** mature gynoecium **C** specimen deposited in herbarium SPSF **D** longitudinal section of flower bud (gynoecium and stamens) **E** immature fruit **F** annular and petiolar scars. Photos: **A**: *Saint-Hilaire s.n* (P00734792); **B**: *Irwin >s.n* (RB161815); **C**: *O.C. Pavão et al.* (SPSF28228); **D**: *R. Marquete 2596* (RB398212); **E–F**: J. C. J. Barbosa.

##### Description.

***Trees*** ca. 20 m tall; ***branches*** cylindrical, with sparse lenticels, glabrous. ***Stipules*** adnate to petiole, 0.5–4 cm long, green, oblong to conical, apex obtuse, base truncate, deciduous, glabrous. ***Petioles*** 2.5–5 cm long, stipular scar along their entire length (100%), glabrous. ***Leaf blades*** 12.7–29.07 cm × 7.8–16.5 cm; ovate-elliptic, apex and base rounded or obtuse, margin entire, papyraceous, venation pinnate, brochidodromous, 8–13 pairs of secondary veins, glabrous. ***Peduncle*** cylindrical, glabrous, annular scars present. ***Flowers*** terminal, solitary, ***flower bud*** ovoid, 3.1 × 3.7 cm, white, glabrous, protected by the perula which is enclosing and protecting the flower bud, perula concave, brownish when dried ; ***outer sepaloid tepals*** 3, 4.5–4.8 cm × 3.5–3.8 cm, broadly elliptic, base truncate, apex apiculate, glabrous, cream-colored; ***inner petaloid tepals*** 6, 3.0–3.8 cm × 2.4–3.2 cm, navicular to obovate, fleshy, base truncate, apex apiculate, cream-colored; ***stamens*** 144–150, 1.2 cm × 0.2–0.3 mm, obovate, spiral arranged in 4 series, thecae 2, introrse, dehiscence longitudinal; ***gynoecium*** 2–3 cm × 2–2.5 cm, hemispherical, cream-colored, carpels 68–71. ***Immature fruits*** 4–8.2 cm × 4.3–8.7 cm, ovoid, brown-green, ***mature fruits*** ca. 17 cm diameter, globose, dehiscence circumscissile, in irregular syncarpous masses, glabrous; ***seeds*** 1–2 per carpel, sarcotesta red.

##### Distribution and habitat.

*Magnoliaovata* is endemic and found in all regions of the country, except the Northeast. It occurs in riparian forest and montane rain forest.

##### Common names.

‘Pinha-do-brejo’: ‘pinha’ means the best-known pine shape like in *Annona*, and ‘brejo’ (swamp) means the habitat where specimens are normally found; Baguaçu.

##### Phenology.

This species was found flowering from September to December, with immature fruits between March and October, and mature fruits between June and September.

##### Preliminary conservation status.

This species has previously been assessed as Least Concern (LC) (CNCFlora 2016). In contrast, in this analysis the area of occupancy (AOO) is about 288.000 km^2^ and it is considered to be Endangered (EN) B2b (i,ii) (IUCN 2022). We need to consider that the knowledge about the genus was scarce at that time. The fact that other authors accepted a broader delimitation of the species and, consequently, a broader distribution for it, impacted their conservation status assessment, which differs from the one recorded in this paper. Despite its wide distribution, the habitat quality of *M.ovata* is not ideal, mainly because there are records in urban areas and without conservation actions.

##### Specimens examined.

**Brasil. Distrito Federal.** Área próxima à Reserva ecológica do IBGE, Cachoeira do Tororó, a ca. de 10 km entrando à esquerda na placa da Fazenda Santa Prisca, 15 Oct 1996, *R. Marquete 2596* (RB398212); Torto, Fundação Zoobotânica, 10 Oct 1961, *E. P. Heringer 6864* (US1691190); Fundação Zoobotânica, 20 Oct 1991, *E.P. Heringer*, *8726* (SP79747) Fazenda água limpa/UnB, mata de galeria do córrego da Onça, coletas efetuadas no final da mata, 7 Jul 1994, *B.M.T Walter 2166* (CEN18475, MBM225747); Estação Ecológica do Jardim Botânico de Brasília, 27 Oct 1964, *I.N.C. Azevedo 204* (HEPH12165; RB210306); Estação Ecológica do Jardim Botânico de Brasília, 7 Nov 2002, *F.P.R Jesus 207* (HEPH121750); Estação Ecológica do Jardim Botânico de Brasília área na borda do projeto Águas do cerrado, 3 Aug 1995, *F. Silva 15* (HEPH12171); Jardim Botânico de Brasília, 23 Sep 2008, *R.C. Martins 100* (HEPH12168); Jardim Botânico de Brasília, 8 Oct 1993, *M. Boaventura 49* (HEPH12159, HEPH8469); Jardim Botânico de Brasília, 29 Apr 1985, *Equipe do Jardim Botânico de Brasília 393* (HEPH12172); Jardim Botânico de Brasília, 20 km de Brasília, 24 Nov 1993, *I.V. Lima 304* (HEPH12169); Mata do Riacho Fundo, Fazenda Sucupira (CENARGEN/EMBRAPA), 18 Aug 1997, *A.B Sampaio 127* (CEN33404); Fazenda Sucupira, mata de Galeria do Riacho Fundo, atrás da churrasqueira, a aproximadamente 5 m da margem direita do Riacho Fundo, 28 Jun 2000, *E.S.G Guarino 250* (CEN39351); Rio Torto, ca. 10 km N of Brasília, 8 Jul 1966 *H.S. Irwin et al. 18092* (SP140657; SP1443714); Reserva Ecológica do IBGE, mata ciliar do córrego Roncador, 5 Jun 1989, *D. Alvarenga & F. C. A. Oliveira 1609* (US3255147); Road Brasília to Taguatinga, forest on marshy ground, 12 Nov 1964, *G.T. Prance >s.n.* (P01753310); Mata do Bananal, atrás da EMBRAPA/CENARGEN, na margem esquerda do Córrego Bananal, 2 Aug 2000, *S. Ernestino et al. 335* (CEN39434); Vicinity of Planaltina, 3 Oct 1965, *H.S. Irwin 8905* (RB210326); **Goiás**: 42 km south of Caiapônia, riverine forest of Rio Claro, 27 Oct 1965, *G.T Prance >s.n* (P01753311); cerca de 2 km após a ponte sobre o rio Preto, sentido Palmital-Cristalina, à esquerda, em frente a entrada da faz. do Sr. Edileno, 11 Sep 2002, *A.A. Santos 1478* (CEN47791); **Mato Grosso do Sul**: Fazenda Panambi, Córrego São Bernardo, 28 Oct 1981, *P.P. Furtado 66* (RB210335); Coxim, Conglomerado, MS-141, Subunidade 01, subparcela 02, indivíduo 18, 12 Apr 2018, *G.H.L Silva 445* (CEN109241); **Minas Gerais**: Serra da Araponga, Fazenda Neblina, 23 Oct 2001, *L.S. Leoni 4755* (RB1341962); Carmópolis de Minas, Estação Ecológica da Mata do Cedro, 11 Dec 2004, *L. Echternacht 778* (HUEFS118654); Serra dos Órgãos, 1 Jan 1839, *Guillermin s.n* (P01753313); Viçosa, ESAV, *Y. Mexia >s.n.* (VIC232); **Paraná**: Antonina Figueira de Braça, 30 Oct 1973, *G. Hatschbach 32972* (MBM31012); Antonina, Rio Pequeno, 18 Aug 1978, *G. Hatschbach 41553* (MBM59973); Antonina, Rio Capivari, 23 Jun 1972, *G. Hatschbach 29731* (MBM37889); Perto da Casa Branca, 10 km W de Cerro Azul, 12 Aug 1966, *J.C. Lindeman 2271* (MBM11594); Cerro Azul, Rib. Do Tigre, 7 Dec 1983, *G.Hatschbach 47636* (MBM88597); Guaraqueçaba, RPPN Salto Morato, trilha do pico, 18 Jul 2013,*M.L. Brotto 1324* (ICN193487; MBM429910); Rio Bananal, 9 Dec 1970, *G.Hatschbach 25776* (MBM22913); Rio do Cedro, encosta de morro, 13 Sep 1967, *G.Hatschbach 17193* (MBM6008); Reserva Natural Salto Morato, Área do Projeto Sucessão, 1 Oct 2001, *F. Putini 2855* (MBM279318); Serrinha, 6 Jul 1967, *G. Hatschbach 16696* (MBM3379); Rio Vermelho, 06 Dec 1972, *G.Hatschbach 30925* (MBM37887); Colônia Parati, 20 Mar 2002, *J.M Silva 3591* (RB210299); Monte Alegre, Embaú, 23 Mar 1954, *J.G. Khulmann >s.n* (RB210320); Jaguariaíva, Rio do Sabia, 28 Nov 1968, *G. Hatschbach 20457* (MBM11348); Morretes, Serra do Marumbi, encosta voltada para América de Cima, 25°28'40"S, 48°53'04"W, 240 m, 11 Jul 2020, *M.L. Brotto 3885* (MBM429910); Porto de Cima, encosta de morro, 4 Jun 1974, *G. Hatschbach 34473* (MBM31011); Marumbi, 16 Nov 1978, *G. Hatschbach 41719* (MBM59972); Porto de Cima, margem do rio, 28 Nov 1973, *G.Hatschbach 33397* (MBM31014); PARNA Saint-Hilaire/Lange, 11 Dec 2017, *R.R. Völtz 1469* (UPCB3822); São João da Graciosa, 07 Nov 1961, *G.Hatschbach 8624* (MBM74971); Paranaguá, Rio Cambará, 24 Oct 1968, *G.Hatschbach 20121* (MBM12255); **Rio de Janeiro**: Petrópolis, Quitandinha, 20 Feb 1948, *O.C. Góes 29* (RB210304): **Santa Catarina**: Barra do Rio do Meio, 14 Mar 2010, *M. Verdi el al. 4475* (FURB23555, JOI6861); Blumenau, Associação Desportiva Hering, Parque da Hering, 31 Jan 2011, *E. Torres >s.n*. (FURB33876); Ilhota, Morro do Baú, 22 Nov 2002, *D.B. Falkenberg 10449* (FURB41585); Jaraguá do Sul, Margem do rio Cerro, 21 Oct 2008, *A. Stival-Santos 148* (FURB8683); Joinville, Piraberaba-Rio da Prata, 17 Oct 2009, *S. Dreveck et al. 1194* (FURB15935); Fortaleza, Praia Grande, 9 Jan 2015, *A.A Oliveira 917* (FURB45398, FURB28181); Praia Grande, 23 Nov 1984, *G.Hatschbach 61236* (HUEFS21717); Pouso Redondo, 11 Nov 2008, *M. Verdi 939* (FURB9484); Rio Esperança, Rio dos Cedros, 8 Dec 2010, *M. Verdi 5949* (FURB32892, FURB28189, JOI15501); Rio Natal, Divisa entre São Bento e Corupá, 25 Nov 2013, *P. Schwirkowski 92* (MBM391903); **São Paulo**: Eldorado, 9 March 1995, *R.R. Rodrigues et al. 161* (ESA026072) Mun. Agudos, Faz. São João do Barreiro, mata de brejo ao lado da represa, 15 May 2012, *G.D. Colletta 653* (ESA118868); Loreto, Araras, 1 Dec 1917, *O. Vecchi >s.n.* (SP1194); Assis, Estação Experimental do Inst. de Agronomia, região alagada, 19 Sept 1989, *J.A Pastore 261* (SPSF13111); Bauru, 27 Oct 2005, *M. Carboni 268* (ESA100050); Bauru, 14 Oct 2005, *M. Carboni 278* (ESA100047); Mun. Buri Estação Experimental de Buri, Floresta paludosa, degradada, 25 Nov 2014, *N.M Ivamauskas 6656* (SPSF49578); Juquitiba, chácara vizinha no Recanto da Paz, 23°58'0"S, 47°6'0"W, 7 Sept 2006, *R.J Polisel 404* (SPSF39085); Piracicaba, 29 Jul 1993, *K.D Barreto et al. 797* (ESA10807); Mun. Pedregulho, Parque Estadual das Furnas do Bom Jesus, em capoeirinha, prox. Casa de Sta. Suzia, 23 Jan 1993, *J.R Guillaumon >s.n.* (SPSF16065); Salesópolis, Bacia de acumulação do Rio Paraitinga, 4 Feb 2001, *S.A. Nicolau 2748* (SP352569); São José dos Campos, 23°04'30"S, 45°56'15"W, mata do Horto, 24 Oct 1985, *A.F. Silva 1327* (VIC10970); São Luiz do Paraitinga, Parque Estadual da Serra do Mar, 2 Dec 2009, *L.S. Silva et al. 1627* (UEC200460); São Miguel Arcanjo, Parque Estadual Carlos Botelho, Área do projeto Parcelas Permanentes, *V.C. Souza el al. 29220* (ESA109549); São Miguel Arcanjo, 13 Mar 2002, *O.T. Aguiar 1105* (ESA104291); São Miguel Arcanjo, Parque Estadual Carlos Botelho, 06 Jan 2015, *B.G. Silva et al. 183* (UEC188962); Serra da Cantareira, 4 Dec 1987, *O.T. Aguiar 221* (SPSF11587); Parque Estadual das Fontes do Ipiranga, Vila Fachini, 13 Aug 1987, *R. Mello-Silva et al. 20* (SP253208); Área da Companhia Votorantim. Estrada entre o alojamento da Barra e a portaria para Tapiraí, 30 Apr 2013, *V.C. Souza 34973* (ESA123872, RB854665, RB854669).

##### Notes.

Several Brazilian *Magnolia* species have been synonymized under *M.ovata*, but one of the main characteristics that differentiate it from the majority of the other taxa is the absence of trichomes in its structures, being the only species native to Brazil without this feature (Table 1). *Magnoliaparanaensis*, previously described as a new species to Paraná, and synonymized with *M.ovata* in the Flora do Brasil (Mello-Silva et al. 2023), does not contain distinguishing features to separate it from *M.ovata*; both have glabrous structures and similar leaf shapes and sizes. The type of *M.paranaensis* was originally identified as *Talaumaamazonica*, but as stated in the abovementioned description of *M.amazonica*, the species can be differentiated by the absence of trichomes in *M.ovata* (vs. trichomes present on the petiole and branches in *M.amazonica*) and the number of carpels: 144–150 in *M.ovata* vs. 98–102 in *M.amazonica*.

One of the morphological characters that most impacts the distinction of *M.sellowiana* and *M.irwiniana* from *M.ovata* is the pubescence of the vegetative and reproductive organs, a characteristic not found in *M.ovata*, which is totally glabrous. Characteristics that can also help when distinguishing these species are the shape and texture of the leaves, in addition to the number of carpels and geographic distribution.

#### ﻿ Magnolia sellowiana

Taxon classificationPlantaeMagnolialesMagnoliaceae

(A.St.-Hil.) Govaerts, World Checklist and Bibliography of Magnoliaceae 72. 1996.

8D5892C8-2C32-5DEF-ACF0-5471031F140B

Figs 6 , 10 , 12

≡ TalaumasellowianaA.St.-Hil., Fl. Bras. Merid. 1:26, pl. 4B. 1824.
≡ Magnoliaselloi Spreng., Syst. Veg., ed. 16 [Sprengel] 4(2, Cur. Post.): 216. 1827.
= Talaumafragrantissima Hook., Ic. Pl. t. 208–212. 1840. Type. BRASIL. Swampy grounds in the Organ mountains, 3000 feet, January 1837, *Gardner 305* (holotype: BM! [BM000574769]).

##### Type.

Brasil. São Paulo: “in sylvis, prope Ipanema, haud longe ab urbe Sorocaba”, fl, *Sellow 2* (lectotype designated here: P! [P00734795]; isolectotypes: F! [F0077437F], P! [P00734796, P00734797], MPU! [MPU027383].

**Figure 10.**
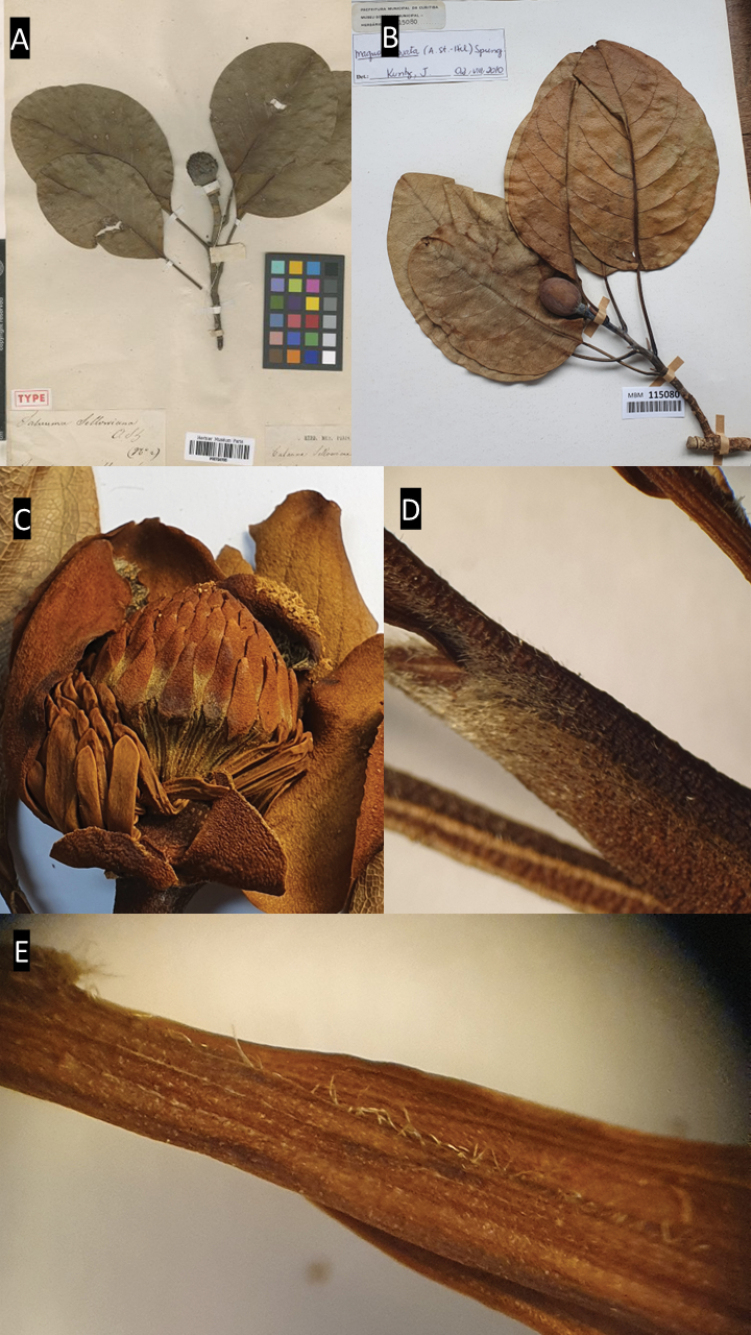
*Magnoliasellowiana***A** specimen deposited in herbarium P showing broadly elliptic leaf **B** specimen deposited in herbarium MBM **C** flower, detail of gynoecium and stamens **D** stipule with trichomes **E** midvein with trichomes. Photos: **A**: *A.Saint-Hilaire s.n.* (P00734795); **B**: *U. Pastore & R.M Klein 145* (MBM 115080); **C–E**: *L.S*. *Leoni 2689* (RB739505).

##### Description.

***Tree*** ca. 15 m tall, ***branches*** cylindrical, with sparse lenticels, with few sericeous trichomes on annular scars, glabrescent. ***Stipules*** adnate to petiole, 0.5–2 cm long, green, oblong to conical, apex obtuse, base truncate, deciduous, tomentose when young. ***Petioles*** 3.7–5,5 cm long, stipular scar along their entire length (100%), tomentose. ***Leaf blades*** 10–17.5 cm × 4.5–10.5 cm, broadly elliptic, base cuneiform, apex rounded or emarginate, entire-irregular margin, papyrus-membranous, young leaves with few trichomes on midvein, glabrescent or trichomes persistent on herbarium material, venation pinnate, brochidodromous, abaxially slightly tomentose when young, adaxially glabrous, 5–13 pairs of secondary veins, glabrous. ***Peduncle*** cylindrical, tomentose at the annular scars, yellow trichomes or glabrescent, annular scars present. ***Flowers*** terminal, solitary, ***flower bud*** not seen; ***outer sepaloid tepals*** 3, 3.4–4.0 cm × 2.7–3.2 cm, navicular to oblong, cream-green, base truncate, apex rounded, fleshy, cream-colored; ***inner petaloid tepals*** 6, 2.7–3.1 cm × 1.5–2.9 cm, cream-colored, obovate to navicular, base rounded, apex rounded, cream-colored, ***Stamens*** ca. 180, 1–1.4 cm × 0.1–0.4 mm, linear, arranged in 8 spiral series, base truncate, apex acute; ***gynoecium*** 1.6–2.5 cm × 1.3–2 cm, hemispherical, carpels ca. 102. Mature ***fruits*** globose, dehiscence circumscissile, in irregular syncarpous masses; ***seeds*** 1–2 per locule.

##### Distribution and habitat.

An endemic species growing in the Southeast (São Paulo, Minas Gerais), and Central-West (Goiás, Mato Grosso do Sul). Found, as most species of the genus in Brazil, in riparian forest.

##### Phenology.

The species was found flowering between March and December and with immature fruits between January and July.

##### Preliminary conservation status.

The species has previously been assessed as Data Deficient (DD) (Khela 2014a). In this analysis, the area of occupancy (AOO) is about 92.000 km^2^ and is thus considered to be Endangered (EN) B2b (i,ii) (IUCN 2022). As a species that occurs in regions like Goiás, which has high rates of forest fires and in regions like São Paulo that suffers from high real estate pressure, *M.sellowiana* needs urgent conservation attention, reforestation in protected areas is suggested.

##### Specimens examined.

**Brasil. Goiás**: Jataí, Sudoeste de Goiás, 11 May 2004, *Souza*, *et al. 3622* (ESA108690); Estrada de acesso à fazenda das Pedras, em frente à sede da fazenda, 16 Jul 1997, *S.P.C. Silva 649* (CEN28390); Ipameri, Fazenda das Pedras, 7 Nov 1996, *S. P. C Silva 500* (CEN30626); **Mato Grosso do Sul**: Botaiporã, Várzea do Rio Samambaia, 7 km L da cidade, 27 Oct 1986, *U. Pastore 145* (MBM115080); Paraná, Município de Sengés, Fazenda Pisa-Papel e Celulose, Poço do Encanto, interior da mata, 18 Dec 1997, *S.I. Elias 306* (ESA377759); Sengés, PCH Fazenda Entre Rios, 26 Mar 2016, *J.M. Silva 9278* (MBM406513); Brasilândia. Estrada Brasilândia- Bataguassu, Córrego Boa Esperança, *A.* 14 Oct 1998, *Amaral Jr. 167* (RB210273, SP334514); Jaguariaiva, Rio Cilada, 18 Feb 1987, G.Hatschbach 50901 (MBM115251); Parque Estadual do Cerrado Jaguariaíva Pr., 10 Oct 2000, *L. von Linsigen 64* (MBM266020); Ventania, Campo de fora, 23 Jul 2004, *D.A Estevan 407* (IAN186917); **Minas Gerais**: Fazenda Neblina-Pq Estadual do Brigadeiro, ao lado da estrada, 2 Apr 1994, *B.S. Leoni 2689* (RB739505); Estação experimental de Café Coronel Pacheco, 5 Sep 1940, *E.P. Heringer 9* (RB44816); Santos Dumont, Posses. Sítio Araçá, Nascentes do córrego Araçá, 27 Mar 2005, *A.P. Fontana 1240* (RB2102370); Viçosa, 12 Nov 1979, *R.S. Ramalho 1659* (RB256157); **São Paulo**: Estrada da Granja TOK, mata na área da bacia de acumulação do Rio Biritiba Mirim, 20 Jan 2001, *S.A. Nicolau et al. 2591* (SP352454); Espraiado, Faz. N. Senhora da Glória, 2 Dec 1935, *J. Mello >s.n* (SP35090); Piracicaba, Rio Claro, Trevo Iracemópolis, 3 Mar 2009, *J. Kuntz 3* (ESA113983); Piracicaba, 3 Mar 2009, *J. Kuntz 2* (ESA113984); Rodovia Piracicaba-Rio Claro-Trevo Iracemápolis, mata de brejo, 9 Oct 2009, *J. Kuntz 4* (RB646302); Rodovia Piracicaba-Rio Claro, Trevo Iracemápolis, 3 Mar 2009, *G.T. Prance 59697* (RB1110753); Mun. de Itapetininga, estação experimental, 29 Nov 1997, *L.C Souza 194* (SP335063, SPSF23732); Itapeva, Estação Experimental de Itapeva, *R.*, 24 Feb 2010, *Cielo Filho 1085* (SPSF43414); Monte Alegre do Sul, 20 Ago 1949, *J.A. Cunha 65* (ESA118919); Monte Alegre do Sul, Bairro do Bugrinho, 20 Jul 1949, *M. Kuhlmann 1809* (SP76739); Penápolis, 20 Ago 1917, *s.c >s.n* (SP439); Queluz, 2 Jul 1899, *s.c 104* (SP23811); Butantã, 4 July 1917, *F.C. Hoehne >s.n* (SP29959); Bois près Hypanema aux environs de Sorocaba Floresta nacional do Ipanema, s.d., *A.Saint-Hilaire s.n* (MO3411335, P00734797).

##### Notes.

**Figure 11.**
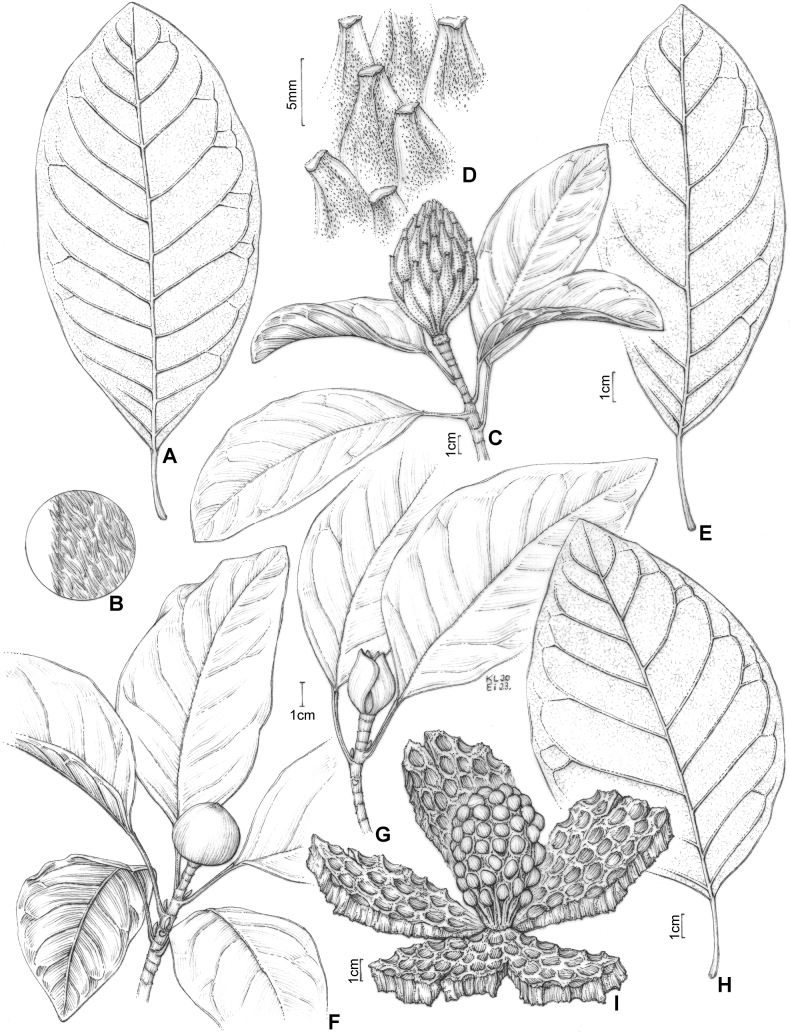
**A** leaf blade *Magnoliaamazonica***B** trichomes from the petiole of *M.amazonica***C***Magnoliabrasiliensis***D** fruit with trichomes in *M.brasiliensis***E** leaf blade *M.brasiliensis***F***Magnoliaovata* showing perule **G** floral bud of *M.ovata***H** leaf blade of *M.ovata***I***M.ovata* mature fruit. (**A–B**: *A.M Barreto 30*; **C–E**: *A.A. Grillo & M. Sztutman >s.n.*; **F–G**: *E.P. Heringer, 8726*; **H**: *R.R. Rodrigues et al. 161*; **I**: based on photographs of J. C. J Barbosa.) Drawing prepared by Klei Souza.

**Figure 12.**
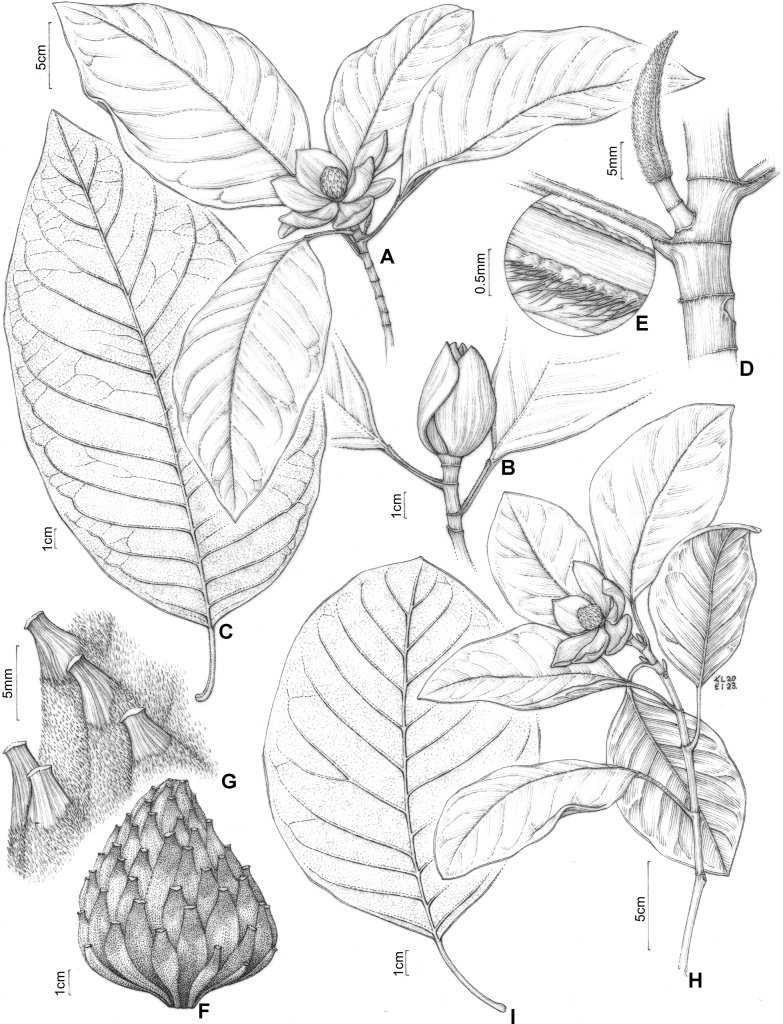
**A***Magnoliairwiniana***B** presence of flower bud in *M.irwiniana***C** leaf blade of *M.irwiniana***D** detail of branch and stipule with trichomes in *M.irwiniana***E** detail of the trichomes on the petiolar scars **F** immature fruit of *M.irwiniana***G** detail of the puberulent trichomes on the fruit **H***Magnoliasellowiana***I** leaf blade *M.sellowiana*. (**A**: based on photographs of D. A. Zavatin; **B**: based on photographs of J.C.J. Barbosa **C–G**: *H.F Leitão Filho et al. 34821*; **H**: based on photographs of D. A. Zavatin; **I**: *M. Kuhlmann 1809*) Drawing prepared by Klei Souza.

*Magnoliasellowiana* is distinguished from *M.ovata* by its broadly elliptic leaf shape, the greater number of carpels (ca. 102), and the presence of trichomes (vs. oval-elliptic leaves, carpels 68–71, and absence of trichomes in *M.ovata*) (Figs 11, 12).

Lozano-Contreras (1990) indicated that one of the P specimens is the holotype, and the remainder the isotype. However, as no details of each specimen are indicated, it is not clear to us which sheet he selected as holotype. Although P00734795 is indicated in the P herbarium database and JSTOR as holotype, we have not found information in the literature that formally proposes this particular sheet as the holotype. Therefore, we have proposed a lectotypification to formally address this issue.

Lozano-Contreras (1990) also mentioned that he had realized that a specimen deposited at P, originally from B, labeled as *Sellow 1*, was identified as *T.ovata*. However, this material is almost identical to the type of *T.sellowiana* and does clearly belong to this species and not to *T.ovata*. The misidentification of *Sellow 1* as *T.ovata* could be what has led authors to consider the two species as identical, and therefore, synonyms.

## ﻿Discussion

The main objective of this study was to present the taxonomic revision of the genus *Magnolia* in Brazil, which had been scarcely documented. For the first time, a thorough taxonomic evaluation has been carried out of the majority of herbarium specimens of native *Magnolias*, collected in Brazil, including type material of all species. Furthermore, targeted fieldwork was conducted, leading to an updated delimitation of the previously accepted taxa for the country and thus changing the number of accepted species for the region. In the context of conservation, these updated species delimitations, based on the morphological study of an extensive number of specimens, are highly significant.

The protologues of most species are short and without much information about the morphological characters. Similar to the descriptions by Lozano-Contreras (1990), we prioritized that the descriptions made here integrated not only the reproductive characteristics but also the vegetative ones, taking into account that the flowers and ripe fruits are difficult to see in herbarium records of *Magnolia* species. Pubescence was a character used, together with reproductive and distribution characteristics, to aid in the separation of species, with trichomes on the fruit being a novel character recognized for *M.brasiliensis*, and that was here described for the first time.

Regarding the synonimization of *M.paranaensis* with *M.ovata*, analysis of specimens in the herbarium and field observations in Paraná supported this decision. The herbarium of the state of Paraná, where the species occurs, was visited, 19 specimens that occur in the region were analyzed, in addition to field work carried out to search for the species. This species was described based on one specimen only, because of the lower carpel number. However, our ongoing research into Neotropical *Magnolias* shows the importance of taking into account a wide range of characters to distinguish between species, in which the carpel number is important but not defining, as it generally concerns a broad range for each species. Hence, it is important to count the number of carpels on many specimens, to be able to include a range rather than a single number. The currently available material for *M.paranaensis* does not allow for that, and therefore, further research is needed to confirm that it is indeed a separate species.

The counting of the number of structures as well as the observation of particular characters need to be carried out during particular developmental phases for both male and female parts. Several chemical studies on the stamens of *Magnolia* species show their importance both in releasing the aroma (Wang et al. 2011) and in understanding stamen development (Nie et al. 2022). It is important to highlight that the stamens of MagnoliasectionTalauma are deciduous in the male phase and shed easily, a known characteristic of the genus that aims to better disperse pollen, being a highly specialized evolution (Figlar and Nooteboom 2004; Canright 1952). The presence or absence of dehiscence of this structure is a taxonomic factor that can separate sections (Kim et al. 2002; Wang et al. 2020). However, quickly shedding structures make accurate counting difficult and, therefore, it is recommended to count the stamens while still in the flower bud.

In fruits, it was not possible in some cases to obtain exsiccates with mature fruit for analysis. Although we can achieve a delimitation using vegetative characters and immature material, it is extremely important to have mature material so that we can analyze the shape of the carpels and characteristics that can change during maturation (e.g., presence of trichomes). It is suggested that a sampling be carried out focused on looking for these characteristics.

Unfortunately, about 190 digital records of Brazilian *Magnolias* did not contain photos of the specimens, and more than about 42,1% (80 specimens) of these could not be identified, mainly for the following reasons: herbarium specimens lack reproductive parts, leaves were crumpled or broken, or reproductive parts were poorly mounted on the specimens, making it impossible to visualize trichomes and carpels. Moreover, about 10 specimens could not be identified, because their characters did not coincide with any of the described native Brazilian *Magnolia*, evidencing that new species may be discovered based on herbarium specimens, and that further exploration in the field is required. These are currently being analyzed for future descriptions. Nevertheless, ca. 300 specimens that could be studied in detail allowed us to present a representative study of the genus in Brazil.

As a consequence of our taxonomic study, five native Brazilian species of *Magnolia* are recognized here and their known distribution areas are updated (Table 1). Prior to this study, two widely distributed native *Magnolia* species were recognized in Brazil, *M.amazonica* and *M.ovata*, both species of Least Concern (Khela 2014; CNCFlora 2016), and are here proposed to be Endangered. The recognition of *M.brasiliensis*, *M.irwiniana* and *M.sellowiana* directly affects knowledge about the distribution and conservation status of *M.ovata*, up to now considered widely distributed. This widespread perception is due to the many herbarium specimens that were misidentified. Many of the previously known locations for *M.ovata* are, in fact, areas where we know instead that *M.brasiliensis*, *M.irwiniana* and *M.sellowiana* occur. Therefore, despite some species having their conservation status published on the IUCN Red List, these assessments did not take into account the number of records that were being collected and recognized as *M.ovata*.

There is currently only a good overview of the population health and threats (e.g. current population trend and continuing decline of mature individuals) of the recently described *M.brasiliensis*. None of the other native Brazilian *Magnolia* species has precise population data, although preliminary fieldwork in (type) localities or areas by the first author of this paper shows that the number of individuals is apparently very low. For instance, in Conceição do Mato Dentro, state of Minas Gerais, only one adult individual of *M.irwiniana* was identified despite the apparent suitability of the habitat. In contrast, in regions like Chapada dos Veadeiros, state of Goiás, and Viçosa, Minas Gerais, several young and adult individuals of *M.irwiniana* and *M.sellowiana* were found in small areas. It is important to note that the presence of a nearby mining company and pipeline may exert ecological pressure on the forest and dispersers.

Studies like this are of utmost importance for the understanding of poorly studied and highly relevant genera such as *Magnolia*. We conclude that not only *M.amazonica* and *M.ovata* do occur in Brazil, but that *M.brasiliensis*, *M.irwiniana*, and *M.sellowiana* are distinct and valid species that should be recognized in this country. This knowledge assisted in assessing the conservation status of each species and understanding the distribution of *M.ovata* throughout the country.

To advance the understanding of the ecology and distribution of species, especially *M.irwiniana* and *M.sellowiana*, which sometimes overlap (in terms of distribution and morphological characteristics), molecular studies are suggested, particularly in population genetics (Aldaba Núñez et al. 2021). The main difficulty in collecting species and attempting to conserve *Magnolia* is the fact that its species are part of threatened, small, fragmented and declining tropical ecosystems. More in-depth molecular studies on genetic differentiation that would help in the analysis of gene flow and possibilities of inbreeding, can help us carry out guided reforestation and the implementation of conservation actions.

## Supplementary Material

XML Treatment for Magnolia

XML Treatment for Magnolia amazonica

XML Treatment for Magnolia brasiliensis

XML Treatment for Magnolia irwiniana

XML Treatment for Magnolia ovata

XML Treatment for Magnolia sellowiana

## ﻿Additional information

### Author ORCIDs

Juliana Cruz Jardim Barbosa https://orcid.org/0000-0002-8753-4915

Maria Beatriz Rossi Caruzo https://orcid.org/0000-0002-8393-214X

Ana Rita G. Simões https://orcid.org/0000-0001-7267-8353

Marie-Stéphanie Samain https://orcid.org/0000-0002-7530-9024

### Data availability

All of the data that support the findings of this study are available in the main text.
